# Generative data augmentation and automated optimization of convolutional neural networks for process monitoring

**DOI:** 10.3389/fbioe.2024.1228846

**Published:** 2024-01-31

**Authors:** Robin Schiemer, Matthias Rüdt, Jürgen Hubbuch

**Affiliations:** ^1^ Institute of Process Engineering in Life Sciences, Section IV: Biomolecular Separation Engineering, Karlsruhe Institute of Technology (KIT), Karlsruhe, Germany; ^2^ Institute of Life Technologies, HES-SO Valais-Wallis, Sion, Switzerland

**Keywords:** chemometrics, convolutional neural networks, process analytical technology, data augmentation, hyperparameter optimization, feature importance

## Abstract

Chemometric modeling for spectral data is considered a key technology in biopharmaceutical processing to realize real-time process control and release testing. Machine learning (ML) models have been shown to increase the accuracy of various spectral regression and classification tasks, remove challenging preprocessing steps for spectral data, and promise to improve the transferability of models when compared to commonly applied, linear methods. The training and optimization of ML models require large data sets which are not available in the context of biopharmaceutical processing. Generative methods to extend data sets with realistic *in silico* samples, so-called data augmentation, may provide the means to alleviate this challenge. In this study, we develop and implement a novel data augmentation method for generating *in silico* spectral data based on local estimation of pure component profiles for training convolutional neural network (CNN) models using four data sets. We simultaneously tune hyperparameters associated with data augmentation and the neural network architecture using Bayesian optimization. Finally, we compare the optimized CNN models with partial least-squares regression models (PLS) in terms of accuracy, robustness, and interpretability. The proposed data augmentation method is shown to produce highly realistic spectral data by adapting the estimates of the pure component profiles to the sampled concentration regimes. Augmenting CNNs with the *in silico* spectral data is shown to improve the prediction accuracy for the quantification of monoclonal antibody (mAb) size variants by up to 50% in comparison to single-response PLS models. Bayesian structure optimization suggests that multiple convolutional blocks are beneficial for model accuracy and enable transfer across different data sets. Model-agnostic feature importance methods and synthetic noise perturbation are used to directly compare the optimized CNNs with PLS models. This enables the identification of wavelength regions critical for model performance and suggests increased robustness against Gaussian white noise and wavelength shifts of the CNNs compared to the PLS models.

## 1 Introduction

Driven by the FDA initiative in 2004 (FDA, 2004), process analytical technology (PAT) has evolved in the past two decades from niche applications to a frequently applied tool widely used in the biopharmaceutical research and manufacturing (Read et al., 2010a; Read et al., 2010b; Ündey et al., 2010; Glassey et al., 2011; Rüdt et al., 2017b; Sauer et al., 2019; Wei et al., 2022; Wang et al., 2022). PAT allows to monitor and control processes efficiently and provides means for real-time release testing or in-process prediction of product quality attributes (Jiang et al., 2017; Markl et al., 2020). Optical spectroscopic techniques such as ultraviolet/visible (UV/Vis), Infrared (IR) and Raman spectroscopy have been shown to enable real-time monitoring across a wide range of pharmaceutical processes (Bakeev, 2005; Feidl et al., 2019; Trampuž et al., 2020; Romann et al., 2022; Rolinger et al., 2023). In combination with multivariate data analysis, these techniques are, e.g., suitable for quantifying product and impurity species from process data (Capito et al., 2013; Brestrich et al., 2016, Brestrich et al., 2018; Rüdt et al., 2017a), identify unknown sample compositions (Liu et al., 2017; Wegner and Hubbuch, 2022), or determine product modifications (Li et al., 2018; Zhang et al., 2019a; Sanden et al., 2019) owing to their fast and non-invasive characteristics and high selectivity in protein analysis.

Current approaches to the quantitative analysis of spectroscopic data heavily rely on multivariate linear regression methods such as partial least-squares regression (PLS) (Banner et al., 2021). Due to the linear behavior, these models typically need a limited number of samples for robust calibration and provide comprehensible metrics for critical model evaluation and interpretation (Wold et al., 2001). Machine learning (ML) methods have gradually been applied to the field of chemometrics and have been shown to sometimes outperform linear methods on various regression and classification tasks, employing artificial neural networks (ANNs) (Long et al., 1990; Santos et al., 2005), Gaussian process regression (GPR) (Cui and Fearn, 2017; Malek et al., 2018), support vector machines (SVMs) (Cui and Fearn, 2017), k-nearest neighbor (kNN) (Wang et al., 2023) or convolutional neural networks (CNNs) (Acquarelli et al., 2017; Bjerrum et al., 2017; Cui and Fearn, 2018; Blazhko et al., 2021; Passos and Mishra, 2021; Rolinger et al., 2021; Wang et al., 2023). Next to the increased accuracy, ML models were found to reduce the amount of preprocessing needed prior to spectral modeling (Cui and Fearn, 2018; Rolinger et al., 2021; Tulsyan et al., 2021; Schiemer et al., 2023) and increase robustness against variability in the data (Cui and Fearn, 2018; Yuanyuan and Zhibin, 2018). Major obstacles to successfully deploy these models for process monitoring in biopharmaceutical operations are the required amount of data for model calibration (Tulsyan et al., 2019; Banner et al., 2021), the high number of hyperparameters (Passos and Mishra, 2022) as well as the necessity for universally applicable diagnostic tools to reduce the black-box character of these models (Burkart and Huber, 2021).

In other branches of ML, where data is more abundantly available, nonlinear methods are in many applications state-of-the-art. Major advances have been made in natural language processing or image analysis by using generative techniques such as data augmentation to further increase the amount and variability of data for building models (Shorten and Khoshgoftaar, 2019; Feng et al., 2021). In Bjerrum et al. (2017), the authors first introduced a data augmentation method used for chemometric CNN models based on simple mathematical modifications of the underlying spectral data to induce artificial offset or slope effects and wavelength shifts. This method was generalized by Blazhko et al. (2021) using the theory obtained from extended multiplicative scatter correction. Both mentioned approaches solely address the variations in the spectral domain and do not extract component-specific information for augmenting experimental data. Other ML approaches have been tested using generative adversarial networks (GANs) (Wu et al., 2021; Mishra and Herrmann, 2021; G. McHardy et al., 2023) or variational autoencoders (VAEs) (Guo et al., 2020), where the different input data are projected onto so-called latent structures before they are recombined into *in silico* representations. Both GANs and VAEs involve neural network structures and hence increase the overall complexity of the approach due to additional hyperparameters. Alternatively, the feature dimension of the experimental data may be extended by stacking the outputs of multiple preprocessing methods as proposed in (Mishra and Passos, 2021d; Passos and Mishra, 2021), however, not addressing the limitation in the number of samples.

Finding the right architecture for the underlying problem and tuning the hyperparameters remains a challenging and laborious task due to a high-dimensional search space and long computation times compared to linear methods (Feurer and Hutter, 2019). While several scholars have proposed rather complex architectures resulting in a large number of trainable parameters (Bjerrum et al., 2017; Liu et al., 2017; Blazhko et al., 2021) for their chemometric CNNs, others chose simple architectures employing solely one convolutional layer to maintain interpretability (Acquarelli et al., 2017; Cui and Fearn, 2018). Automating the process of architecture search and hyperparameter tuning, which is commonly referred to as hyperparameter optimization (HPO), reduces the amount of manual work needed to build ML models and helps to identify the best overall configuration. Model-based HPO methods such as Bayesian optimization have been shown to be more efficient at finding the global optimum for computer vision (Bergstra et al., 2013) and chemometrics (Passos and Mishra, 2021, 2022; Rolinger et al., 2021) compared to randomized or grid-based approaches.

While linear methods such as PLS are well understood and many evaluation metrics exist to assess model quality, ML models are often considered black boxes due to the increased amount of parameters and different mathematical principles. For CNNs, various visualization methods exist to understand the trained convolutions and the corresponding feature importance (Zeiler and Fergus, 2013; Yosinski et al., 2015). Gradient-weighted class activation maps (GradCAMs) as proposed in Selvaraju et al. (2020) have already been applied to chemometrics (Mishra and Passos, 2021b; Passos and Mishra, 2021) to provide quantitative insights into the contributions of a specific wavelength. However, GradCAMs are not directly comparable to conventional evaluation metrics for PLS models such as regression coefficients or otherwise computed PLS-specific importance metrics. Additive feature attribution methods such as Shapley additive explanations (SHAP) (Lundberg and Lee, 2017) or Shapley additive global importance (SAGE) (Covert et al., 2020a; Covert et al., 2020b) provide model-agnostic frameworks to compute quantitative feature importance based on multivariate permutations.

In this manuscript, we develop and implement a novel data augmentation method for generating synthetic spectral data based on the local estimation of the pure component profiles. We further establish a holistic modeling workflow for chemometric data considering data augmentation, HPO, and interpretation. The herein calibrated CNN models are evaluated using three different data sets from protein chromatography employing UV/Vis spectroscopy as well as one publicly available data set using IR spectroscopy. Firstly, the suitability of the proposed data augmentation method to enlarge small experimental data sets is demonstrated and a systematic tuning of the method is performed. Secondly, the optimal configuration of the CNN model is determined by automated HPO. Thirdly, we assess the interpretability of the optimized models by quantification of the importance of individual wavelengths. Finally, the robustness and transferability of the optimized CNNs are studied by *in silico* perturbations and model transfer to external data sets.

## 2 Materials and methods

**FIGURE 1:**
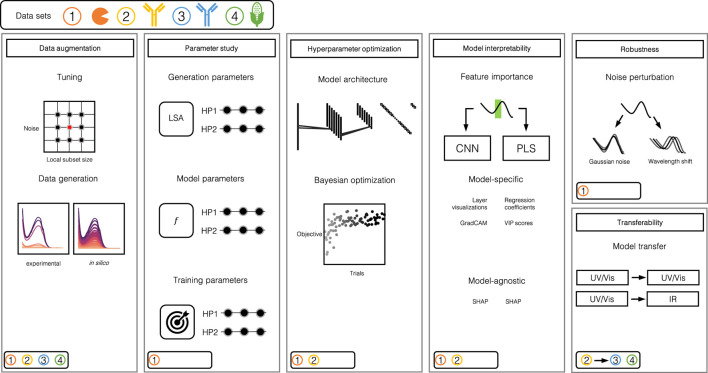
Illustration of the study design. The study can largely be divided into 5 steps: 1) Data augmentation, 2) parameter study, 3) automated HPO, 4) model interpretability assessment and 5) robustness and transferability evaluation. The round boxes in the bottom left corners indicate which data set was used for which step. Data set 1 consists of three model proteins, namely Rib A, Cyt C, Lys. Data set 2 and 3 are comprised of mAb size variants and data set 4 stems from samples of corn.

The evaluation of chemometric CNNs in this manuscript involves multiple steps which are performed on the basis of four data sets. Figure 1 provides an illustrative overview of the individual steps and the data sets used within each step. In this section, the methodology for the individual steps is explained in detail.

### 2.1 Data and equipment

In this study, four data sets based on spectroscopic data were used for the evaluation of the herein-presented methods. Data sets 1–3 originate from chromatography experiments of which the experimental details were presented elsewhere (Brestrich et al., 2016; Brestrich et al., 2018). Data set 4 was presented in (Blazhko et al., 2021). A summary of the experimental conditions and the data subsets reserved for training and testing of the developed models are given in Supplementary Table S1. For data sets 1–3, the training and test sets were chosen as presented in the referred literature, where the rationale was to evaluate the trained models on independent chromatography experiments with varying process conditions. For data set 4, a random split was used as no additional information about the underlying experiments was available. In the following, we will refer to all data points in a data set as samples. One sample consists of an absorption spectrum and the corresponding concentration values obtained by fraction analytics. In data sets 1 to 3, all samples stem from fractions of chromatography elution peaks.

#### 2.1.1 Data set 1

Experimental procedures for data set 1 can be found in (Brestrich et al., 2016). The data set consists of 233 samples stemming from five chromatography experiments with varying elution conditions. From each experiment, fractions were collected and analyzed for the concentrations of the three protein components ribonuclease A (Rib A), cytochrome C (Cyt C), and lysozyme (Lys). The experiments were monitored by UV/Vis spectroscopy using a wavelength range of 240 nm–300 nm at a resolution of 1 nm resulting in 61 features for regression modeling. This data set involves well-studied model proteins and therefore serves for method development within this study.

#### 2.1.2 Data set 2

Data set 2 consists of 432 samples stemming from four chromatography experiments with varying elution conditions. The experimental procedures for the data set can be found in (Brestrich et al., 2018). The concentrations of monoclonal antibody (mAb) monomers and aggregates were obtained by fraction analytics. For UV/Vis monitoring, a variable path length spectrometer was used operating at a wavelength range of 240 nm–340 nm at a resolution of 2 nm, resulting in 51 features for regression modeling.

#### 2.1.3 Data set 3

Experimental procedures for data set 3 can be found in (Brestrich et al., 2016). The data set consists of 348 samples stemming from three chromatography experiments with varying elution conditions. The concentrations of the mAb size variants low molecular weight species (LMWS), monomers, high molecular weight species (HMWS)1, and HMWS2 were obtained by fraction analytics. The experiments were monitored by UV/Vis spectroscopy using a wavelength range of 240 nm–300 nm at a resolution of 1 nm, resulting in 61 features for regression modeling.

#### 2.1.4 Data set 4

Data set 4 was obtained from (Blazhko et al., 2021) and consists of in total of 80 samples from IR spectroscopy. The data was obtained from analyzing corn samples and the contents of oil, protein and starch were given as reference values. The spectral range is 1,100 cm^−1^ to 2,500 cm^−1^ at a resolution of 2 cm^−1^, resulting in 701 features for regression modeling. The training and test subsets were assigned using a randomized 80:20% split.

#### 2.1.5 Hardware and software

Data analysis was done in Python 3.8. Data augmentation was performed using *numpy* (v. 1.19.5), *scikit-learn* (v. 1.1.1) and *scipy* (v. 1.7.3). CNNs were implemented in *tensorflow* (v. 2.5.0). HPO was done in *optuna* (v. 3.1.0) in connection with a MySQL^TM^8.0 database and *PyMySQL* (v. 1.0.2). SHAP values were computed using *shap* (v. 0.41.0). All computations were done using a workstation equipped with AMD Ryzen 9 3900X 12-core processor and 32GB of memory operating Microsoft Windows 10.

### 2.2 Data augmentation

**FIGURE 2:**
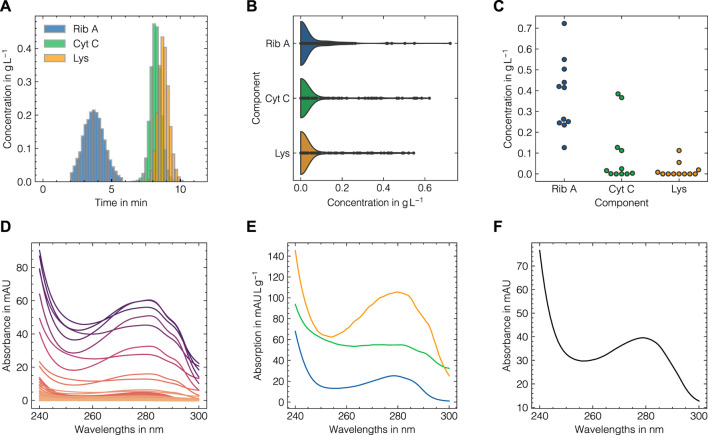
Visualization of the LSA data augmentation method. The method is based on experimentally derived response data **(A)** and spectral data **(D)**. Firstly, the response data distributions are approximated by kernel density functions **(B)**. Second, for a specific sample for which a new spectrum is generated, vector distances for all instances in the supplied experimental data are computed. The *n*_LSA_ nearest samples in terms of most similar concentration profiles are then selected **(C)** to form a representative subset of the sampled concentration vector and posing as the basis to derive the pure component spectra **(E)**. Given the sampled concentration vector, the pure component spectra are combined into a newly generated sum spectrum **(F)**.

Before describing the data augmentation method mathematically, the motivation is laid out. In spectroscopy, each molecule is considered to possess a unique spectrum characterized by well-defined extinction coefficients. However, in practical scenarios, various factors such as detector saturation, noise, wavelength shifts, or interfering buffer species can influence the observed absorption spectra. The proposed data augmentation method aims to incorporate these effects by local approximations of the pure component profiles. The method can largely be divided into three consecutive steps: 1) Concentration density approximation, 2) subset selection, and 3) spectra generation. An illustrative overview of the data augmentation method is presented in Figure 2. Mathematically, a given data set can be described as $X,Y=\left\{\left(x_{i}^{T},y_{i}^{T}\right)\right\}$ for *i* ∈ [1, *M*] with *M* being the total number of samples and $x_{i}^{T}\in R^{1xN}$ and $y_{i}^{T}\in R^{1xP}$ being absorbance spectra with *N* wavelengths and concentrations of *P* components, respectively. First, the value distribution in **Y** is approximated for each column using a kernel-density estimation as implemented in *scipy.gaussian_kde*. From the approximated distribution, a random concentration vector $y_{*}^{T}$ is sampled. The distance between the sampled vector $y_{*}^{T}$ and all instances in **Y** is then computed according to Eq. (1)

$$d\left(y_{*}^{T},y_{i}^{T}\right)=\|y_{*}^{T}-y_{i}^{T}{\|}_{l},\;for\;i\in \left[1,M\right]$$ (1)

with *l* being the order of the vector norm. As a second step, a number of *n*_LSA_ samples with the smallest values $d\left(y_{*}^{T},y_{i}^{T}\right)$ are selected to form a local subset of available samples with matrices $\tilde{X},\tilde{Y}$ . In the third step, pure-component profiles are estimated based on these local subsets by solving the linear problem as given by

$$\tilde{X}\tilde{S}=\tilde{Y},$$ (2)

where $\tilde{S}\in R^{NxP}$ are the estimated pure-component profiles for the local subsets $\tilde{X},\tilde{Y}$ . The solution of (2) is realized by a ordinary least-squares or non-negative least squares (NNLS) solver to constrain solutions to positive values as implemented in *numpy* or *scipy*, respectively. Thirdly, an *in silico* spectrum x_*_ is calculated by Eq. (3)

$$x_{*}=\tilde{S}y^{*}.$$ (3)

The motivation behind assembling a subset of samples with closely similar compositions lies in the pursuit of extracting the local differences in the pure component estimations between experimental data points, e.g., induced by concentration differences or higher noise contents. By focusing on samples that are closely related in composition, we aim to enhance the quality of the *in silico* spectra. As the generation of *in silico* spectra is based on local subsets of the available data, the method is coined local subset augmentation (LSA). A more detailed explanation of the approach can be found in the Supplementary Section S1. To add more variation to the synthesized spectrum, a Gaussian white noise distributed as $N\left(0,\sigma_{\text{noise}}\right)$ is added to each feature and a normally distributed wavelength shift $N\left(0,\sigma_{\text{shift}}\right)$ is applied to the entire spectrum $x_{*}^{T}$ . In summary, the LSA method presents several configurable parameters, namely *n*_LSA_, *l*, *σ*_noise_, *σ*_shift_, and the type of solver employed to derive the pure-component profiles. These so-called hyperparameters are automatically tuned using a cross-validation scheme. The residuals between the *in silico* and the measured spectra were used as quality metrics and summarized by the root mean squared error (RMSE) of reconstruction. Hyperparameters associated with the LSA method were screened using a grid-based scheme and the optimal configuration with regard to the cross-validated reconstruction error RMSECV was selected. Depending on the data set, the determined local subset size served as an initial estimate and was further refined within the optimization procedure described in Section  2.3.3.

### 2.3 Convolutional neural networks

CNNs are multivariate regression models, which may be composed of several convolutional, pooling, fully connected (FC), dropout, and regularization layers. For a comprehensive, theoretical overview of CNNs the reader is referred to pertinent literature (Goodfellow et al., 2016; Rosebrock, 2018).

#### 2.3.1 Neural network architecture

The following design choices in neural network architecture were made based on existing studies (Cui and Fearn, 2018; Rolinger et al., 2021; Passos and Mishra, 2022), while aiming to keep model complexity low in order to reduce computational time during HPO. It is worth noting that the UV/Vis data mostly used in this study usually is of lower dimension than Raman or IR spectroscopy data used in other studies and hence the required model complexity may be lower. CNNs were constructed from 1 to 3 convolutional layers. For each convolutional layer, a number of 1–10 convolutional filters with a customized filter width were defined. After the first and second convolutional layers, a maximum pooling layer was implemented using a window size of 2, effectively halving the number of features generated from the previous layer. As the convolutional filter width is constrained by the output dimension of the previous layer, the maximum allowed filter width is adjusted accordingly after each pooling step. After the convolutional block, a flattening operation was implemented to concatenate the outputs from all convolutional filters of the last convolutional layer into a one-dimensional vector. In the regression block, an FC layer with up to 100 units was used. The output layer is configured to use a rectified linear unit (reLU) activation function to restrict the prediction to positive values. As activation functions for the convolutional and FC layers, linear and hyperbolic tangent (tanh) functions were used, respectively. Other options may individually be chosen and several options were tested within this study. Depending on the chosen architecture, the number of configurable hyperparameters may greatly vary and hence a standardized workflow for optimization is required. The base architectures and corresponding hyperparameters for training and data augmentation used for data sets 1 and 2 are listed in Table 1.

**TABLE 1:** Overview of hyperparameters used for data augmentation, CNN architecture and training for data sets 1 and 2.

Category	Hyperparameter	Base	Data set 1		Data set 2	
			Initial	Optimized	Initial	Optimized
Data augmentation	Number of gen. Samples	1e5	1e5	1e5	1e5	1e5
	Local subset size	5	11	11	13	12
	Distance norm	2	2	2	1	1
	Solver type	NNLS	NNLS	NNLS	NNLS	NNLS
	Std. White noise	0.001	0.001	0.001	0.001	0.001
	Std. Wavelength shift	0.01	0.01	0.01	0.03	0.03
Model architecture	Number of convolutional layers	1	1	2 (1–3)	1	3 (1–3)
	Number of conv. Filters	5	5	[2, 7] (1–10)	5	[2, 10, 8] (1–10)
	Filter width	9	9	[3, 9] (3–61)	9	[7, 5, 11] (3–51)
	Pooling width	2	2	2	2	2
	Number of FC units	12	12	29 (5–100)	12	9 (5–100)
	Activation function conv. Layer	linear	linear	linear	linear	linear
	Activation function FC units	sigmoid	tanh	tanh	tanh	tanh
	Initialization function weights	glorot uniform	random uniform	random uniform	random uniform	random uniform
	Dropout rate	0	0	0 (0–0.3)	0	0.07
	Regularization factor	0	0	0 (10^−9^–10^−3^)	0	3.07 × 10^−9^ (10^−9^–10^−3^)
Model training	Learning rate	10^−3^	10^−3^	10^−3^	10^−3^	10^−3^
	Batch size	100	100	100	100	100
	Optimizer	Adam	Adam	Adam	Adam	Adam
	Patience	4	4	4	4	4

The base configuration refers to hyperparameter values used during the single-factor parameter study. Data augmentation parameters were derived from tuning of the LSA method and a simple CNN architecture was assumed. The initial configuration refers to settings adapted after the parameter study and served as a comparison for the optimized models. The optimized configuration refers to values derived from HPO. Here, the square brackets indicate the optimized parameter values. Multiple numbers are given for the determined values for the individual layers. The parenthesis denote the search spaces during HPO. If no search space is given, the parameter was not included in the optimization. For the convolutional filter width, the allowed maximum filter width was configured to halve with each additional layer due to the interposed pooling layers.

#### 2.3.2 Training, cross-validation and testing

In the context of training CNN models, data produced by the LSA method are denoted *in silico* data, while experimental data are split into training and test data as listed in Supplementary Table S1. For the remainder of the manuscript, we will further refer to *calibration* as the process of fitting the CNN model weights and *training* as an entire cycle of generating *in silico* data, fitting the weights, and evaluation based on the training data.

To train a CNN model, the *in silico* subset is generated solely based on the training data, and the CNN model is solely calibrated on the *in silico* data. For cross-validation, a similar data setup was used. Here, the experimental training data is rotated in a *leave-one-group-out* scheme, i.e., holding out one of the assigned training experiments. During each rotation, the experimental training data are split into rotation-specific training and test sets. Again, the *in silico* data are generated based on the rotation-specific training data and are further used to calibrate the CNN model. In both cases, training and cross-validation, the assigned training data serve to evaluate the stopping criteria. The CNN models were calibrated for a maximum of 100 epochs using the mean squared error (MSE) of all responses as the loss function and the stochastic gradient-based optimizer referred to as Adam (Kingma and Ba, 2015). For all parameter studies and HPO, the number of generated samples was set to 10^5^, and model calibration was stopped when the loss of the assigned validation set did not improve for 4 consecutive epochs further referred to as *patience*. Finally, the CNN models were evaluated on the independent test set, which has not been used for *in silico* data generation or cross-validation.

To study the effect of hyperparameters associated with data generation, CNN architecture, or training on model performance, hyperparameters were varied in a one-factor-at-a-time scheme while all other parameters remained constant. Model performance was measured using the cross-validation error RMSECV across all response variables and the optimal settings were adapted as the base configuration for subsequent HPO. This parameter study was solely conducted with data set 1. The findings for the initial configuration were then also used for HPO for data set 2.

#### 2.3.3 Hyperparameter optimization

HPO routines were implemented in *optuna* for data sets 1 and 2. For both data sets, a combination of a randomized and a tree-parzen estimator (TPE)-based sampler was used. A random sampling of hyperparameters in pre-configured ranges was performed for the first 100 trials when the optimizer switched to the TPE. The TPE can be used to optimize continuous, discrete, and categorical variables at the same time using a Bayesian approach based on kernel-density estimations. For the technical details and the theory of the method, the reader is referred to Bergstra et al. (2013); Akiba et al. (2019). The sum of the component-specific cross-validated coefficients of determination $\sum_{i=1}^{P}R_{\text{CV, i}}^{2}$ was used as the objective value. Automated pruning of unpromising hyperparameter combinations was configured to set in after 100 trials and was triggered when $\sum_{i=1}^{P}R_{\text{train, i}}^{2}$ was lower than the median of all previously reported trials. This effectively reduces the computation time as cross-validation does not need to be performed for the pruned trials. The search spaces for the optimizer were determined based on previously conducted individual parameter studies and are listed in Table 1. A MySQL^TM^  database was used to facilitate distributed computation to accelerate HPO. In total, the optimization was run over 500 trials. However, the effective number of finished trials differs due to automated pruning.

Among the top 5 models, the best candidate was selected based on quantitative metrics such as training and cross-validation performance as well as qualitative metrics such as model complexity. The selected model was retrained for 10 repetitions using a modified patience of 10. The obtained performance metrics were compared with optimized PLS models as measured by the normalized error $NRMSE=RMSE/\bar{y_{i}}$ with $\bar{y_{i}}$ being the arithmetic mean of the observed concentration for the respective data subset. As a second baseline comparison, the optimized CNN models were trained without any prior data augmentation. Therefore, the models were trained for 300 epochs and the early stopping criteria were disabled. The training data were divided randomly into 80/20% calibration-validation subsets to determine after which epoch the best performance was achieved.

### 2.4 Partial least squares regression

PLS models were implemented in *scikit-learn* using the non-linear iterative partial least squares (NIPALS) algorithm. While the CNNs were used as multi-response models, i.e., predicting all target species using the same model, single-response PLS models were used for each component. Spectral data were preprocessed using a Savitzky-Golay filter (SGF) and mean-centered. HPO for PLS models was performed using a grid-based scheme. Therefore, the number of PLS components (1–10), the order of derivative (0–2) and the width of the smoothing window of the SGF (3–31) were varied in pre-configured ranges as stated in parenthesis. The SGF was used with a second-degree polynomial. The optimal configuration was chosen using the cross-validated and scaled sum of squared errors SSECV_scaled_ according to Wold et al. (2001) as given by Eq. (4)

$${SSECV}_{\text{scaled}}=\frac{\sum_{i=1}^{M}{\left({\hat{y}}_{i}-y_{i}\right)}^{2}}{M-n_{\text{PLS}}-1},$$ (4)

where ${\hat{y}}_{i}$ and *y*_*i*_ denote the predicted and observed response values for a sample *i*, respectively, and *n*_PLS_ designates the number of PLS components.

### 2.5 Feature importance

To quantitatively evaluate the importance of individual wavelengths, GradCAM and SHAP were employed. While GradCAM can solely be applied to the CNNs, SHAP is model-agnostic and can therefore be used to directly compare CNN and PLS models. For the PLS models, the regression coefficients and variable importance in projection (VIP) scores were used as evaluation metrics. Feature importance techniques were solely employed using the optimized models from HPO for data set 1 and 2.

#### 2.5.1 Gradient-weighted class activation maps

Guided GradCAM is a response-discriminative localization technique which was proposed in Selvaraju et al. (2020) and was implemented according to Rosebrock (2018). GradCAM can be largely divided into three steps: 1) Computation of backward gradients with respect to each response variable and the last convolutional layer for one specific input spectrum, 2) global average pooling of the computed gradients along the wavelength dimension to obtain a single weighting value for each filter in the last convolutional layer, and 3) computation of the GradCAM estimate. In cases, where pooling layers are used in between convolutional layers, the localization estimate is of reduced dimension compared to the original input spectrum and is thus linearly interpolated to match the original dimension.

#### 2.5.2 Shapley additive explanations

SHAP is a model-agnostic additive feature attribution method derived from economic game theory and can be used to quantify feature importance which are in turn referred to as SHAP values. To compute a SHAP value for a specific wavelength, the absorbance values in a given data set are randomly permuted and replaced by absorbance values sampled from a conditional distribution. Every sample in the given data set is permuted *d* times and passed through the regression model to obtain the model prediction for the permuted input spectrum. The permuted model prediction *w*(*S*) is compared to the prediction using the original data *w*(*S* ∪ {*i*}). The SHAP value *ϕ*_*i*_(*w*) for a feature *i* and a permutation cycle *w* with *d* permutations (*w*_1_, …, *w*_*d*_) is according to Lundberg and Lee (2017) then defined as Eq. (5)

$$\phi_{i}\left(w\right)=\frac{1}{d}\underset{S\subseteq D\backslash \left\{i\right\}}{\sum }{\left(\begin{array}{c} d-1\\ \left|S\right| \end{array}\right)}^{-1}\left(w\left(S\cup \left\{i\right\}\right)-w\left(S\right)\right)$$ (5)

where *D* and *S* denote the entire feature set and the permuted feature subset, respectively. To incorporate inter-dependency due to collinearity between multiple wavelengths, conditional sampling is performed for multiple wavelengths at the same time and repeated for a fixed number of permutations. For a deeper overview of the theory and discrepancies to closely related methods, we refer to (Lundberg and Lee, 2017; Covert et al., 2020b; Covert et al., 2020a; Belle and Papantonis, 2021). To compute SHAP values within this study, the test subsets for data sets 1 and 2 were used to obtain permuted input spectra. All computations were done as implemented in *shap* using the *PermutationExplainer*. In total, 10^5^ permutations per input spectrum were used.

#### 2.5.3 Variable importance in projection

The VIP scores are a common metric to assess variable importance in PLS models next to the regression coefficients. According to (Mehmood et al., 2012), the VIP score *v*_*j*_ for a wavelength *j* ∈ [1, *N*] is defined as Eq. (6)

$$v_{j}=\sqrt{N\sum_{a=1}^{A}\left[\left(q_{a}^{2}t_{a}^{T}t_{a}\right){\left(w_{aj}/\left\|w_{a}\right\|\right)}^{2}\right]/\sum_{a=1}^{A}\left(q_{a}^{2}t_{a}^{T}t_{a}\right)},$$ (6)

where *w*_*a*_, *q*_*a*_, and *t*_*a*_ denote the loading weights, the y-loadings and the scores vector corresponding to the PLS component *a* ∈ [1, *A*], respectively. The total number of wavelengths is given by *N*.

### 2.6 Robustness and transferability

The robustness and transferability of the LSA method and the optimized CNN were evaluated by an *in silico* noise perturbation study and a model transfer to two external data sets based on UV/Vis and IR spectroscopy.

#### 2.6.1 *In silico* noise perturbation

To compensate for increasingly noisy data, both model types were evaluated with modified generated data sets with an increasing level of white noise $N\left(0,\sigma_{\text{noise}}\right)$ with *σ*_noise_ of {0.001, 0.005, 0.01, 0.05, 0.1, 0.5, 1} mAU and an increasing level of axial wavelength shifts $N\left(0,\sigma_{\text{shift}}\right)$ with *σ*_shift_ of {0, 0.05, 0.1, 0.15, 0.2, 0.25, 0.3} nm. We differentiate between models solely being trained on the noise-free data set and models being retrained for each noise level. The latter models are referred to as “retrained.”

#### 2.6.2 Model transfer to data set 3

To evaluate model performance on a data set without additional HPO, the optimized model from data set 2 was transferred to data set 3. The CNN input and output dimensions were adjusted according to the experimental data. The CNN models were trained in the optimized configuration for 10 repetitions and evaluated against the PLS models which were optimized as described in Section 2.4.

#### 2.6.3 Model transfer to data set 4

To compare the LSA method to the augmentation method presented in Blazhko et al. (2021), which will be referred to as extended multiplicative scatter augmentation (EMSA), both methods were applied to data sets 2 and 4. The CNN architecture found for data set 2 was therefore transferred to data set 4 without additional HPO. To account for the higher dimension of the IR data, the pooling window size of the first CNN layer was adjusted to 4 and the number of FC units was raised to 25. The EMSA method was obtained from Blazhko et al. (2021) and used with its default configurations. The LSA method was tuned as described in Section 2.2. IR spectra were preprocessed using a second derivative SGF with a window size of 19 and second-order polynomial, as this was reported to improve the performance of the EMSA method (Blazhko et al., 2021).

## 3 Results

### 3.1 Generating highly realistic *in silico* spectra from experimental data

**FIGURE 3:**
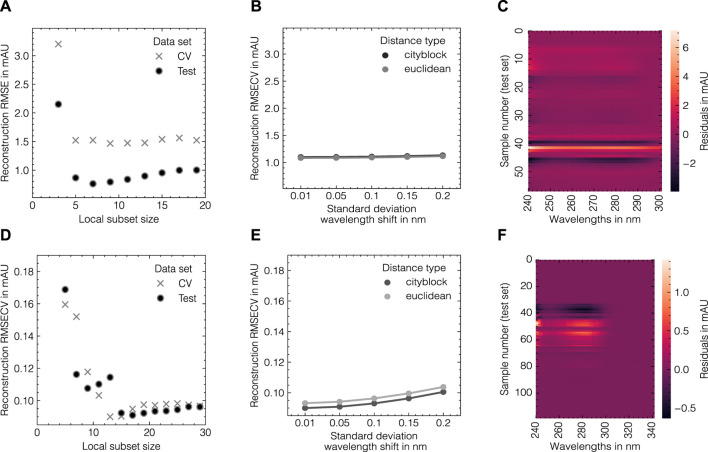
Tuning of the LSA data augmentation method for data set 1 **(A–C)** and data set 2 **(D–F)**. From left to right, the reconstruction RMSE in dependence of the local subset size, the reconstruction RMSE in dependence of the standard deviation of the wavelength shift and a heatmap of wavelength-specific residuals for the respective test subsets are shown. The errors in **(A, B, D, E)** are shown for fixed levels of white noise *σ*_noise_ = 0.001.

**FIGURE 4:**
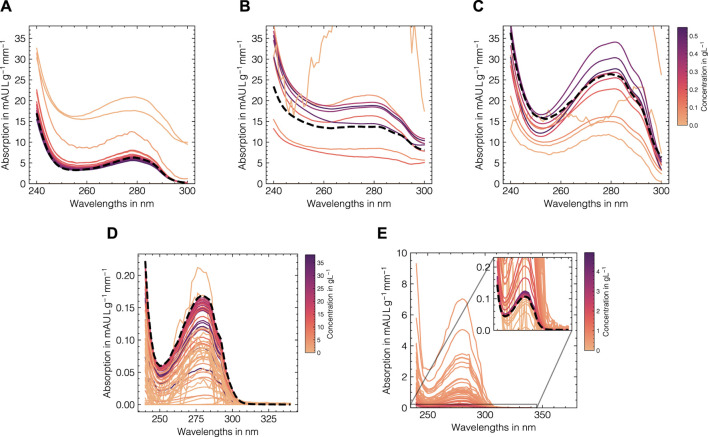
Local estimations of the pure component profiles for Rib A **(A)**, Cyt C **(B)**, Lys **(C)** for data set 1 and monomer **(D)** and aggregate **(E)** species for data set 2. The pure component spectra are shown for each sample in the test data (solid lines) with a local subset size of 11 for the data augmentation method and are colored according to the concentration of the corresponding component with darker colors denote high concentrations. The pure component spectra estimates using the entire training data are shown as dashed black lines.

The LSA method was used to generate *in silico* UV/Vis absorbance spectra based on the assigned training data as previously described in Section 2.2. To systematically evaluate the suitability of the proposed data augmentation method and tune the corresponding hyperparameters, the LSA method was used to reconstruct the experimental data in a cross-validation scheme. The reconstruction accuracy for the cross-validation and test subsets for both data sets as measured by the RMSECV are displayed in Figures 3A, B, D, E with regard to the local subset size and the standard deviation of the applied wavelength shift. The local subset size strongly affects the reconstruction RMSECV and shows optima at sizes of 5 and 13 samples for data sets 1 and 2, respectively. In both cases, the RMSECV remains stable for small wavelength shifts and grows exponentially starting at 0.1 nm. The Cityblock norm is observed to slightly improve the RMSECV for data set 2 compared to the Euclidean norm while the overall impact on RMSECV is considerably small in comparison to the local subset size. While the Cityblock norm uses absolute differences, the Euclidean norm is based on squared differences and hence can affect the selection of local subsets depending on the concentration ranges in the samples. The residuals for the spectra in the test sets are displayed in Figures 3C, F and show maximal deviations of 5 and 15% for data sets 1 and 2, respectively, as measured by the maximum deviation normalized by the maximum absorbance in the corresponding run. As LSA is based on local subsets of the experimental data, the pure component profiles differ depending on the selected data points. Figure 4 shows the local pure component profiles for all components from data sets 1 and 2 for concentration samples stemming from the test data. The color of the lines indicates the concentration of the respective component in the corresponding sample. The dashed lines indicate the global pure component profiles using the entire training data for estimation instead of the local subsets. For all components, the local profiles are scattered around the global profiles with larger deviations for samples in extraordinarily high or low concentration regimes. Particularly for data set 2, these effects are visible for the aggregate component, where the local pure component profiles strongly deviate from the global estimates for low concentration regimes by a factor > 10. Contrarily, for the monomer species low concentration regimes cause the spectra to capture an increased level of noise in the data.

In summary, LSA provides a concentration-adaptive data augmentation method by leveraging variations in the spectral and concentration domain. The tuned LSA method can generate highly realistic *in silico* spectra and can hence be used to augment experimental data sets.

### 3.2 Training convolutional networks with augmented spectral data

**FIGURE 5:**
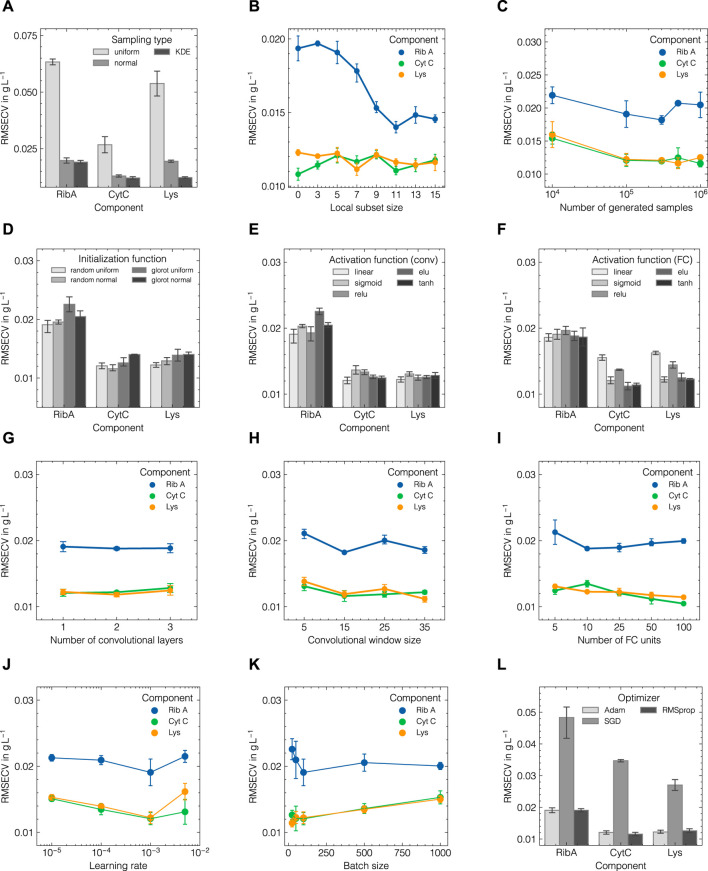
Influence of selected hyperparameters on the predictive cross-validation performance (RMSECV) for each component in data set 1. The selected hyperparameters can be categorized in three groups: generation parameters **(A–C)**, model parameters **(D–I)** and training parameters **(J–L)**.

To study the effect of hyperparameters associated with data augmentation, CNN training, and model architecture on predictive performance, hyperparameters were varied in a one-factor-at-a-time scheme while all other parameters remained constant. The base configuration of the CNN for this parameter study can be found in Table 1. The obtained performance as measured by the predictive RMSECV for all response variables for data set 1 are displayed in Figure 5.

Considering the data augmentation hyperparameters in Figures 5A–C, the kernel-density estimation (KDE) sampling surpasses the performances obtained by uniform and normal sampling. The local subet size shows a stable performance for Cyt C and Lys between 5 and 15 samples, while the RMSECV for Rib A is reduced by approximately 25% by increasing *n*_LSA_ from 5 to 11, reaching a more balanced performance between all three components. This suggests that it may be beneficial to include the local subset size during HPO to ensure finding the optimal solution for all response variables. Hence, the local subset size was subsequently incorporated during HPO for both data sets 1 and 2. The number of *in silico* generated samples positively affects model performance and starts to plateau at 10^5^. As increasing the number beyond that only minorly affects model performance while increasing computational time considerably, 10^5^ was adopted for the base configuration for HPO.

The influence of initialization and activation functions for convolutional and FC layers are shown in Figures 5D–F. Initialization only minorly affects model performance with random uniform providing the best option. While the linear function performs best for the convolutional layer, non-linear activation functions show superior accuracy for the FC layers with tanh and the exponential linear unit (ELU) returning the lowest RMSECV values for all components. Increasing the complexity of the CNN by increasing the number of convolutional layers, the number of FC units, or the size of the convolutional window, does not directly improve model performance. Performance gains are not equally distributed among all response variables and no overall trends can be extracted as depicted in Figures 5G–I. Regarding the training parameters, it is suggested that a learning rate of 10^−3^, a batch size of 100, and the Adam optimizer provide the best options as shown in Figures 5J–L.

In summary, it was possible to identify optimal configurations for hyperparameters for data augmentation and model training. However, given the high dimensionality of the search space of the remaining hyperparameters and unequally distributed effects on model performance it is suggested to use automated HPO to identify the optimal model architecture for each data set individually.

### 3.3 Automating hyperparameter search by Bayesian optimization

**FIGURE 6:**
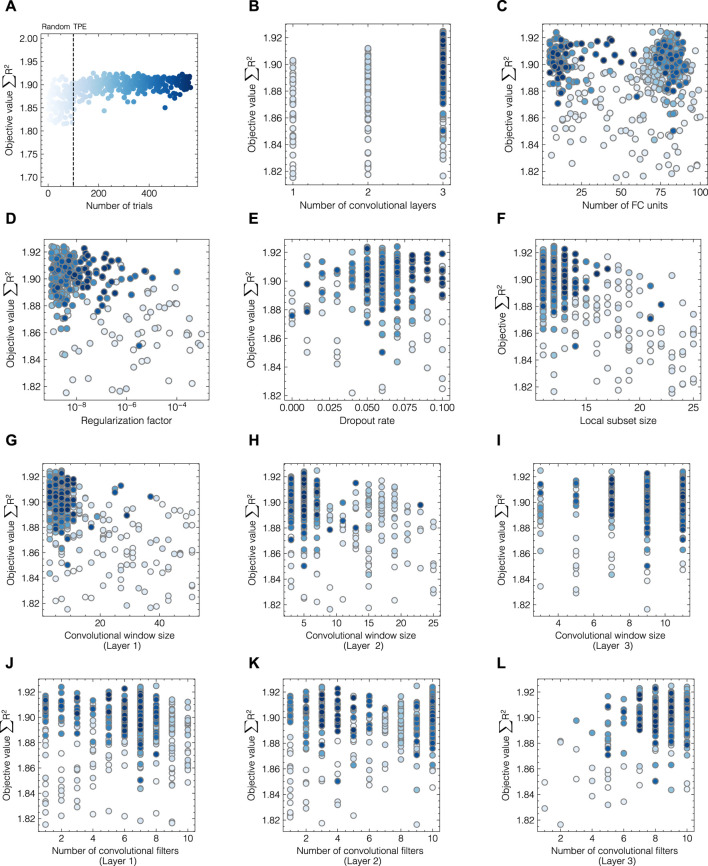
Hyperparameter evolution profiles during optimization for data set 2. The objective values *∑R* ^2^ are shown for all trials **(A)** and each hyperparameter individually **(B–L)**. The colors of the circles indicate the number of HPO trials with darker shades of blue corresponding to later sampling points. Random sampling was performed for the first 100 trials after which median pruning and the TPE-based optimization were enabled.

As hyperparameter search is a multi-dimensional, computationally expensive problem, automated HPO was performed using a TPE-based Bayesian optimizer as implemented in *optuna*. The optimizer considers local and global hyperparameters which enables the solution of optimization problems with multiple decision levels such as the choice of the number of convolutional layers and the optimization of a set of layer-specific hyperparameters. Figure 6 presents the evolution of objective values with regard to all studied hyperparameters throughout the optimization process for data set 2 exemplarily. As shown in Figure 6A, the transition between random and TPE-based sampling can be captured after 100 trials. The objective values form a band centered around 1.9 with scattered maxima at 1.93, with 2 being the maximum achievable objective value. The color of the points indicates the number of the optimization trials with dark blue being the end of the optimization process. The evolution profiles of global parameters (cf. Figures 6B–F) suggest an optimum at 3 convolutional layers with less than 25 FC units. Both regularization methods, governed by the regularization factor and the dropout rate, were found to positively influence model performance although weight regularization using a fairly low regularization factor in the range of 10^−8^–10^−6^ was employed in later trials. In contrast to data set 1, where the local subset size needed to be adjusted for optimal model performance, here, the previously determined value was found to be suitable for the given data set and only varied slightly between 9 and 15 in later trials. According to Figures 6G–L, the three-layer CNN achieves the highest accuracy for all components with a convolutional window size between 1 and 10 for all three layers. The number of convolutional filters shows no clear optimum for the first and second layers, while higher counts are found beneficial for the third layer.

For data set 1, optimal performance with an objective value close to 2.98 was achieved by employing a two-layer CNN with 2 and 7 convolutional filters, a window size of 3 and 9, and 29 FC units. Contrarily, dropout and regularization were both found to be disadvantageous. The evolution plot can be found in the Supplementary Figure S1. The exact hyperparameters and visualization of the architecture for both optimized models can be found in Table 1 and Supplementary Figure S2, respectively.

**FIGURE 7:**
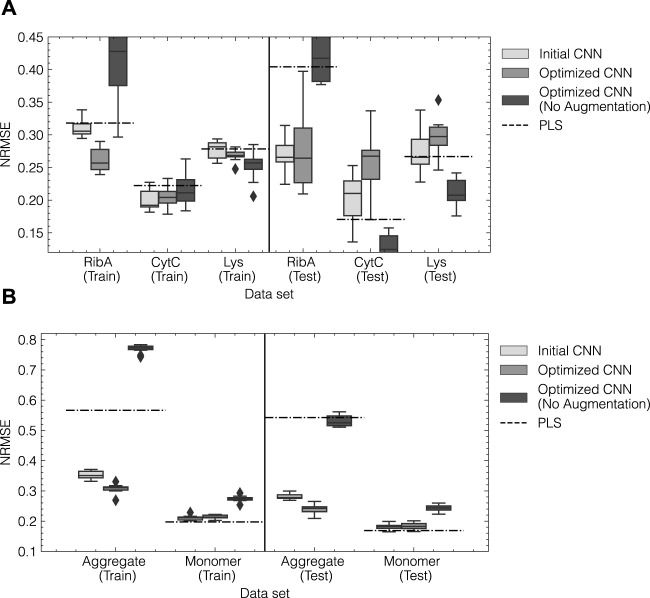
Model performance for initial and optimized CNN models for data set 1 **(A)** and data set 2 **(B)**. The normalized error NRMSE for training and test data are shown on the left and right, respectively. For both data sets, the initial and optimized CNN model were trained 10 times with an elevated patience of 10. The arithmetic mean of the obtained performances is indicated by the solid lines within the boxes. Outliers are shown as diamonds and were defined as such when the error exceeded 1.5 times the interquartile range. The dashed lines correspond to the NRMSE obtained from an optimized PLS model using a SGF for preprocessing as a benchmark.

The optimized CNN models were retrained for 10 repetitions using random initialization of the weights and an increased patience of 10 epochs. The obtained model predictions for training and test subsets for both data sets are summarized by the NRMSE and presented in Figure 7. The boxplots show the distribution of errors for the 10 repetitions as a result of random initilizations and the stochastic nature of the training process. CNN models using the initial configuration obtained from tuning the augmentation method are also included for reference. As a baseline comparison, the NRMSE obtained from optimized single-response PLS models are indicated by the dashed lines. Optimized PLS hyperparameters can be found in Supplementary Table S1. Additionally, the optimized architectures were trained without using any prior data augmentation as described in Section 2.3.3. For reference, timely predictions for the optimized CNN and the PLS can be found in Supplementary Figure S3.

For data set 1 (cf. Figure 7A), initial and optimized CNN models show generally lower NRMSE on average than the PLS models for the training subset for all components. In the test subset, the prediction error for Rib A is reduced by up to 50%, while the test errors for Cyt C and Lys increase. This increase can be attributed to erroneous predictions of Cyt C and Lys during the elution of Rib A as can be seen in the timely predictions in Supplementary Figure S3. The initial CNN architecture performs slightly better on the test subset than the optimized CNN architecture as indicated by a lower average and variance. Interestingly, CNN models without prior data augmentation perform similarly to the PLS models achieving higher accuracy for Cyt C and Lys and significantly lower accuracy for Rib A compared to the CNNs using data augmentation. For data set 2, the optimized CNN model reduces the NRMSE compared to the initial architecture and the PLS model by up to 50% for the aggregate species. The accuracy for the monomer species slightly decreases compared to the PLS model with a 5% increase in the NRMSE. The reduction for the aggregate species can be attributed to the improved capture of the onset of the elution peak as can be seen in the timely prediction profiles in Supplementary Figure S3. In contrast to data set 1, the optimized CNN without augmentation results in considerably higher NRMSE than both augmented CNN models and the PLS. In summary, HPO enabled the automated identification of the optimal model architecture for data sets 1 and 2, leading to improved quantification of Rib A and mAb aggregates, respectively, while sometimes reducing the accuracy for the other species compared to the benchmark methods.

### 3.4 Understanding model predictions through feature importance

**FIGURE 8:**
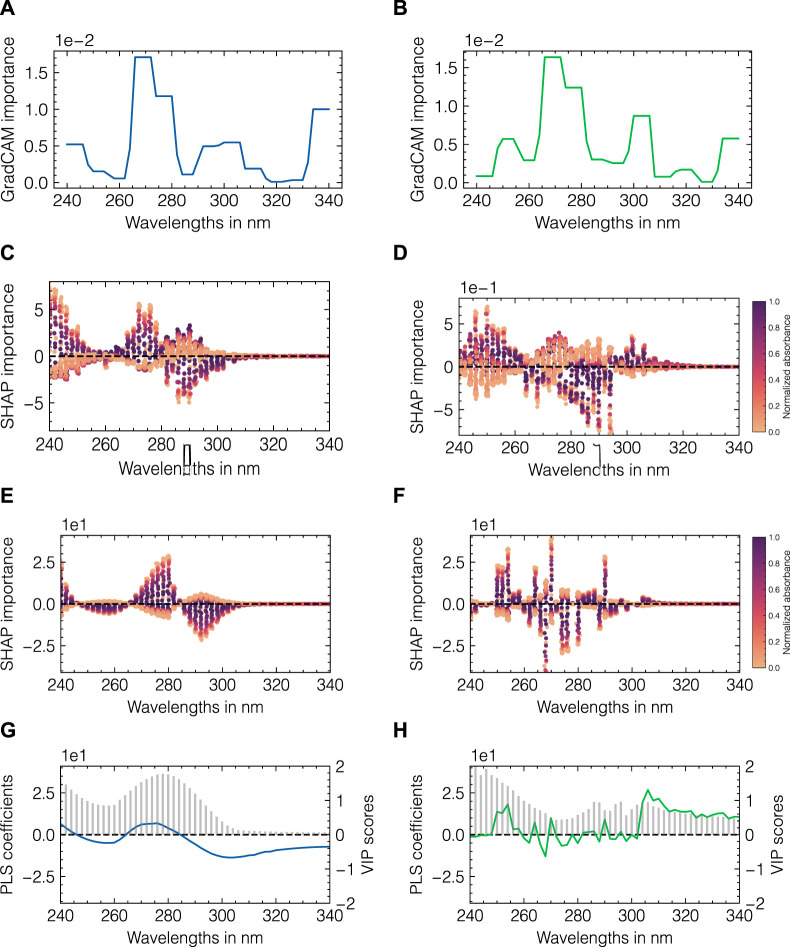
Model-specific and model-agnostic importance measures for data set 2. CNN-specific GradCAM importance values **(A, B)**, SHAP values for CNN and PLS **(C, D)** and **(E, F)**, respectively. Each dot in **(C–F)** corresponds to the feature contribution for one specific model output in the test data set, which are colored according to the measured absorbance. The absorbances were normalized by the maximum value at 280 nm. PLS-specific regression coefficients (shown as lines) and VIP scores (shown as bars) are presented in **(G, H)**. The left and right columns correspond to importance metrics for the monomer and aggregate species, respectively.

To investigate the differences in model performance, model-specific and model-agnostic importance metrics were used. Figure 8 presents an overview of multiple importance metrics for data set 2. CNN-specific GradCAM localization maps are shown in Figures 8A, B. PLS regression coefficients and VIP scores are shown in Figures 8G, H. To enable a direct comparison between the two model types, SHAP values are illustrated in Figures 8C–F. The left and right columns correspond to the feature importances for the monomer and aggregate components, respectively. For the monomer species, both CNN- and PLS-SHAP values closely resemble the PLS regression coefficients with wavelengths between 260 and 280 nm positively contributing to the model output and wavelengths between 280 and 300 nm being assigned negative values. A similar behavior can be observed for the GradCAM estimates with both wavelength ranges being assigned positive importance as GradCAM is constrained to positive values per definition. Additionally, GradCAM identifies the border areas at the beginning and the end of the spectrum as important. The SHAP values generally confirm those observations for small wavelengths, whereas wavelengths above 315 nm are shown to not affect the model output for both the CNN and PLS models. This is in accordance with the PLS-VIP scores as well as the spectral data as no absorbance is detected at wavelengths above 315 nm (cf. Supplementary Figure S4). For the aggregate species, GradCAM provides a fairly similar profile compared to the monomer species showing a shift in the importance peak from 280–305 to 300–305 nm. The PLS regression coefficients support this observation with a maximum of 315 nm for the aggregate species, while the VIP scores for all wavelengths between 280 and 340 nm are between 0.5–1. Regression coefficients for smaller wavelengths appear comparably noisy and without any structural integrity in contrast to the coefficients for the monomer species. While CNN-SHAP values indicate a similar profile as seen for the monomer with an inversed importance for wavelengths between 295 and 305 nm, PLS-SHAP suggests a high degree of noise and considerably lower importance for the previously identified wavelength region relative to the remainder of the spectrum. As a means for comparison, the visualization of the convolutional layers for data set 2 can be found in Supplementary Figure S5. For each convolutional layer and each corresponding filter, the convoluted input spectra for the test set are shown. To emphasize the differences between the monomer and aggregate components, the samples with the maximum concentration of monomers and aggregrates are marked in green and blue, respectively. While the convoluted signals in layer 1 largely resemble the original input spectra, more fluctuations between positive and negative outputs are introduced with layers 2 and 3, with some filters showing fairly similar profiles within one layer. Due to the interposed pooling layers, the resolution of the presented profiles is gradually decreasing across layers 1 to 3.

In summary, the employed feature importance methods point to an elevated importance for higher wavelengths for the aggregates compared to those for the monomer species and identify this area as critical for differentiation between both species. The filter visualization further provides insights into the working principle of the CNNs. Analogous to the presented comparison for data set 2, the feature importance evaluation and filter visualization for data set 1 can be found in Supplementary Figures S6, S7, respectively.

### 3.5 Evaluating robustness and transferability

**FIGURE 9:**
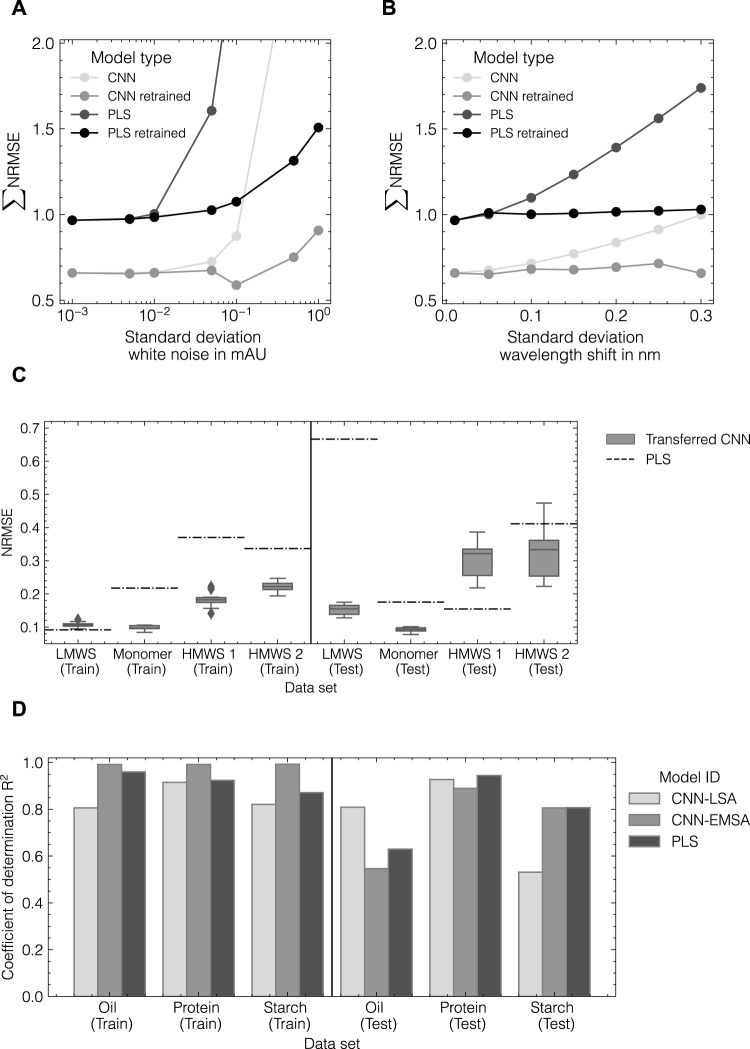
Evaluation of CNN robustness and transferability. Comparison of CNN and PLS model robustness for increasing noise **(A)** and wavelength shifts **(B)** in spectral data for data set 1 as measured by the normalized error NRMSE. CNN performance after model transfer to data set 3 **(C)**. Comparison of model performance with data augmentation conducted by the EMSA and LSA method for data set 4 **(D)**.

To evaluate the capability of the CNN and PLS models to compensate for increasingly noisy data, both model types were retrained using modified generated data sets with 1) an increasing level of Gaussian noise and 2) an increasing level of axial wavelength shifts. The model performance as summarized by the sum of NRMSE for all three components in data set 1 is shown in Figures 9A, B. It is observed that the PLS model does generally not reach the same level of accuracy as the CNN model being trained on the generated data. For both cases, 1) and 2), the PLS model error starts to increase at lower levels of white noise and wavelength shift than the CNN model. Similarly, when both models are retrained at each noise level, the CNN model is observed to be more robust against noisy data and can slightly decrease the model error at a noise level of 0.01 mAU or a wavelength shift of 0.03 nm.

Using the hyperparameters determined during HPO for data set 2, the CNN was trained on data set 3 including data augmentation by the LSA method to assess the generalizability of the identified architecture. All hyperparameters were obtained from data set 2 and remained unchanged. Figure 9C presents a comparison of CNN and PLS models as measured by the NRMSE. The transferred CNN achieves NRMSE values lower than 0.25 for all components for the training subset and decreases the observed errors for the monomer, HMWS1 and HMWS2 species by up to 50%. For the test subset, the variance of the transferred CNN increases and the error for HMWS1 increases compared to the PLS model. The PLS model fails to predict the LMWS component which was observed to depend on the cross-validation split used for optimizing the PLS models.

Finally, the herein-developed LSA data augmentation method was compared to the EMSA method (Blazhko et al., 2021). Both methods were evaluated based on data sets 2 and 4 to assess the applicability of EMSA on UV/Vis data as well as the transferability of the LSA method to IR data. Therefore, the optimized CNN model from data set 2 was trained with *in silico* data from the LSA and the EMSA method for both data sets 2 and 4. A detailed overview of the results for both data sets including the performance of PLS models can be found in Supplementary Table S3. Figure 9D provides a summary of the obtained performances using the LSA method versus the EMSA method for data set 4. The LSA method in combination with a three-layer CNN could successfully be transferred to data set 4 using IR data. When compared with the EMSA method as employed in Blazhko et al. (2021), the LSA method achieves accuracies of +32%, +4%, and −34% in terms of the relative difference in *R* ^2^ for oil, protein, and starch content in the test subset, respectively. However, the training accuracy for the CNN-EMSA model is considerably higher for all three components indicating potential overfitting of the model. When applied to data set 2, the EMSA method fails to generate high-quality data to be used for chemometric modeling resulting in a two-fold increase in RMSE for both components compared to the LSA method.

In summary, the optimized CNN models in combination with the LSA method present a more flexible alternative to commonly used PLS models due to their increased robustness against noisy data and good generalization capability to other UV/Vis spectroscopy data. They may further be universally applied to other types of spectroscopic data without prior HPO.

## 4 Discussion

### 4.1 Data augmentation

Data augmentation can be used to enlarge experimental data sets by extracting and recombining information from the collected data. This is considered to avoid overfitting and thus improve the generalizability of ML models (Shorten and Khoshgoftaar, 2019). The herein presented LSA method builds upon this idea and leverages latent spectral information in terms of pure component profiles. By employing a local subset selection, these pure component profiles are locally approximated and flexibly adapted to the concentration regime of the sample composition for which a new spectrum is generated. The local subset selection further induces the mixing of spectral information from different experimental runs. We consider the designed working principle beneficial to leverage locally available spectral information. While other methods solely take variations in the spectral domain into account, e.g., by modifying the spectra based on variations in model coefficients (Bjerrum et al., 2017; Blazhko et al., 2021), the LSA method projects the information to a latent space in the spectral domain and also accounts for variations in the concentration domain. This idea is similar to algorithms employed in variational autoencoding or GANs (Guo et al., 2020; Mishra and Herrmann, 2021), while maintaining interpretability by employing simple numerical methods. By using a cross-validation-based reconstruction approach, we showed that the LSA method can be tuned by various hyperparameters and is able to reconstruct the hold-out test set of the experimental data with a maximum error of roughly 5% and 15% for data sets 1 and 2, respectively. While pure component profiles for samples within the center of the experimental data range showed slight variations around the global estimates, samples with exceptionally low or high concentration regimes were shown to incorporate more noise or considerably diverge from the global estimates. The shape and magnitude of the estimated profiles is dependent on the selected subsets. For low-concentrated samples, the selected spectra exhibit a low signal-to-noise ratio potentially causing distortions in the spectral shape or numerical inflation of the estimated profiles. Whereas for high-concentrated samples, the density of similar data points is lower resulting in local adaptations of the pure component profiles. In the case of data set 2, there are several samples almost solely containing monomer or aggregate species due to the working principle of the underlying separation process the data is taken from. When one component concentration is close to 0, the estimation algorithm will numerically inflate the component’s profile and hence a higher absorption is observed. The described handling of different concentration regimes is considered to introduce more variance in the *in silico* data. Although the estimates obtained for the extreme data points are not realistic, their impact is considered to be low. It was further observed that the local subset size determined by the tuning method did not provide the optimal value in the case of data set 1. This may be explained by the low absorbance signal by Rib A causing a reduced influence on the reconstruction error compared to Cyt C and Lys (Hansen et al., 2011; Rüdt et al., 2019).

When comparing the LSA and the EMSA method for training CNNs, it was observed that CNN-EMSA models tend to overfit the training data, potentially caused by solely depending on variations in the spectral domain. Whereas the CNN-LSA model resulted in consistent training and test performances. When applied to UV/Vis data, the CNN-EMSA models were not able to achieve the same level of accuracy as the CNN-LSA models. As the scatter correction method, on which EMSA is based, is routinely used with vibrational spectroscopy data (Martens and Stark, 1991; Afseth and Kohler, 2012), the lower accuracy may be explained by insufficient accuracy of the estimated model or an overestimation of the variations in the UV/Vis spectra. However, it should be noted that the EMSA method was used with its default configuration (Blazhko et al., 2021) and may potentially be tuned to be applicable to UV/Vis data.

Many studies published in the literature do not use any type of data augmentation before chemometric modeling with CNNs (Acquarelli et al., 2017; Cui and Fearn, 2018; Malek et al., 2018; Zhang et al., 2019b). This can either be realized by using comparably large data sets in combination with simple model architectures, e.g., in Cui and Fearn (2018) or by longer training on the same data (Zhang et al., 2019b). For the herein evaluated data sets, training CNNs without prior data augmentation resulted in unbalanced model performance with regard to all components which was comparable to the performance of the PLS model. On the one hand this suggests that non-linear models such as CNNs do not necessarily improve the accuracy compared to PLS models. In theory, the CNNs without augmentation have little leverage over PLS models as they are potentially overparameterized given the number of available training data. On the other hand this suggests that data augmentation effectively helps to extract more information from the data to enable more accurate multi-response modeling. This may especially be beneficial in cases where one of the target components contributes a comparably weak spectral signal or when all target components exhibit highly similar spectral profiles. While data set 1 comprises proteins with clearly differentiable UV/Vis absorbance spectra (Hansen et al., 2011), the size variants of a mAb are more challenging to distinguish (Brestrich et al., 2018). Based on the given results, we hypothesize that by augmenting spectral data, minor differences in spectral profiles or signals from underrepresented components can be amplified and therefore increase regression performance. Other spectroscopic methods such as IR or Raman can provide higher selectivity depending on the analytes and hence the benefits of data augmentation should be studied in more detail for those spectroscopic methods. Blazhko et al. (2021) pointed out that for the classification of yeast and mould species using IR data, the usage of data augmentation positively affected the classification performance.

In conclusion, the combination of CNNs and data augmentation via the LSA method provides a flexible, generally applicable approach for chemometric modeling of multiple quality attributes. Given the findings mentioned above, it would further be interesting to study the effect of spectral mixing introduced by the LSA method with data sets considerably larger than the ones used within this study, possibly including data from multiple spectrometers, target proteins, or cell lines.

### 4.2 Model architecture and hyperparameter optimization

Finding the optimal configuration for complex ML models such as CNNs is challenging due to the high-dimensional search space and potentially long computation times (Feurer and Hutter, 2019). In this study, automated HPO based on Bayesian optimization has been used to optimize CNN configurations for data sets 1 and 2. Here, the aim was to maximize the predictive accuracy of the CNN models, while keeping model complexity low. To weight all response variables, i.e., molecular species, equally, the optimizer used the sum of *R* ^2^ of the cross-validation residuals. While equal weighting of all components may also be realized by other evaluation metrics, the sum of *R* ^2^ was chosen to provide a simple figurative metric. During the optimization, the structure of the neural network was varied simultaneously with other hyperparameters such as the local subset size *n*_LSA_ as well as regularization parameters, namely the regularization factor and the dropout rate. In general, increasing the number of convolutional layers implicitly increases the number of hyperparameters to optimize as each layer is assigned an individual set of parameters. At the same time, the intermediate number of features is reduced by the pooling layers thus potentially reducing the number of units in the FC layer.

For data set 1, the identified architecture using two convolutional layers was found to achieve only marginally improved performance compared to the initial configuration. For the second data set, the optimized configuration could improve the initial configuration considerably with regard to the contaminating aggregate species. The ineffectiveness observed for data set 1 may be caused by a suboptimal splitting of the experimental runs or non-exhaustive exploration of the search space as well as potential overfitting on the training subset. It should however be noted that the achieved accuracy lies above 0.97 in terms of the *R* ^2^ for all components and the differences between the studied models are considered marginal. In the context of chemometrics, TPE-based Bayesian optimization has previously been applied to the HPO of CNNs (Passos and Mishra, 2021; Passos and Mishra, 2022). While previous studies have used pruning and optimization based on the simple training-validation splits, the herein-used cross-validation approach is considered more robust and less prone to overfitting (Rolinger et al., 2020). A collection of more detailed practical remarks for using HPO can be found in Supplementary Section S2.

Furthermore, it is unclear from previous studies which hyperparameters should be included during HPO. While Passos and Mishra (2021) vary the depth of the regression block by incorporating more FC layers, an increased number of convolutional layers was found beneficial within this study. Additionally, it was found that critical hyperparameters requiring optimization are the number of filters in each convolutional layer, the convolutional window size per layer, and the number of FC units in the regression block. Increasing the number of filters increases the model’s capacity to learn complex features from the input data. However, a larger number of filters potentially requires a higher number of FC units and thus can cause slower training time (Szegedy et al., 2015). The convolutional window size determines the size of the receptive field, which affects the model’s ability to capture relevant features from the input data (Goodfellow et al., 2016, chap. 9). Smaller sizes capture local features, while larger sizes capture more global features (Gu et al., 2018). Here, the optimal window sizes were found to be as small as 3 for the first convolutional, with a general increasing trend for additional layers. With regard to CNN design choices, multiple suggestions have been made in literature including simple architectures with only one convolutional layer (Acquarelli et al., 2017; Cui and Fearn, 2018), or more complex models using multiple FC layers and a comparably high number of convolutional kernels (Bjerrum et al., 2017; Passos and Mishra, 2021, Passos and Mishra, 2022). Whereas, others adapted design choices made for widely applied computer vision architectures such as the so-called vgg-block design (Blazhko et al., 2021) or inception modules (Zhang et al., 2019b) or even tried to combine the convolutional block with other types of regression models such as Gaussian process regression (Malek et al., 2018). Given the variety of studied architectures, it is difficult to draw a conclusion on which architecture works best for which type of spectroscopic data as they are often combined with prior preprocessing. Transferring the model architecture identified for data set 2 to data sets 3 and 4 in this study, was found suitable for modeling UV/Vis and IR spectroscopic data. The CNN with three convolutional and one FC layer, using weight and dropout regularization, was shown to work well with previously unknown data sets. Especially for data sets with few available experiments or samples, the transfer of CNN architectures is preferred over potentially unstable HPO. It has been shown that multiple factors may affect the successful transfer of chemometric CNNs (Mishra and Passos, 2021c; Mishra and Passos, 2021a), leading to a high degree of freedom in HPO.

While HPO has shown great potential for improving the performance of chemometric models in this study and several other cases (Brunel et al., 2021; Passos and Mishra, 2021), further studies are needed to investigate which CNN architectures are the most suitable for different types of spectroscopic data such as UV/Vis, IR, and Raman spectroscopy. In this regard, the authors advocate for a large-scale HPO study using multiple data sets at the same time. Such research could help identify the most viable CNN architectures for chemometrics in terms of accuracy, generalizability, and robustness, and pave the way to dedicated pre-trained models for biotechnological applications. Pre-trained models facilitate easier transfer from case to case by computationally less expensive transfer learning (Mishra and Passos, 2021c).

### 4.3 Model interpretability

Explainability of machine learning models has become an important topic of research in recent years (Belle and Papantonis, 2021; Burkart and Huber, 2021). In the context of spectroscopy, it is critical to understand which elements of the spectral data are rendered important and contribute to the model’s predictions. In this study, an interpretability assessment compared the predictions of CNNs with PLS models. Four feature importance metrics were used to compare the models: Guided-GradCAM, SHAP values, PLS regression coefficients, and PLS VIP scores.

The results for data set 2 showed that three of four methods were able to identify a small wavelength region that was relevant for differentiating between the two target components. For data set 1, the distinction between the three target components was less pronounced as the importance profiles for Cyt C and Lys were found to be fairly similar for CNN and PLS models. For Rib A, it was possible to resolve clear differences between both model types which supports the improved performance of the CNN model. In general, the studied methods differ in their level of directness in assessing the importance of individual wavelengths. Established methods such as GradCAM or other visualization methods for CNNs (Zeiler and Fergus, 2013) are generally applicable to all types of model architectures (Selvaraju et al., 2020) and enable a fast inspection of the chemometric model. In other studies (Mishra et al., 2021; Passos and Mishra, 2022), it has been shown that GradCAM was able to identify the most suitable section of features from a preprocessing-based extension method. However, as the variable importance is computed from the feature maps of the final convolutional layer, it is directly influenced by previous normalization and pooling operations causing lower resolution profiles or potentially distorted peak locations (Blazhko et al., 2021). Similar to GradCAM, PLS VIP scores provide a positively constrained importance metric. In combination with the PLS regression coefficients, the VIP scores are a commonly applied metric to evaluate the chemical information used by the PLS model (Goldrick et al., 2020; Wei et al., 2022) or conduct variable selection (Mehmood et al., 2012). While the regression coefficients display positive and negative attributions, the VIP scores help determine which variables mostly contribute to the prediction. Despite their practicality, the VIP scores are specific to PLS models and do not contain any information about the variable sensitivity.

Other chemometric studies exist where SHAP estimation was used to identify the most important variables for various chemometric models (Haghi et al., 2021; Mahynski et al., 2022; Guindo et al., 2023). By using SHAP values in this study, it was possible to identify the most relevant features in a direct comparison with the PLS model. Using SHAP or other closely related feature removal-based importance quantification techniques is computationally more expensive than analyzing regression coefficients or GradCAM importance (Covert et al., 2020a). Especially for spectroscopic data, where multiple wavelengths are highly correlated, permutation-based feature importance techniques should be handled carefully by ensuring a sufficient number of permutations and permuting a coalition of wavelengths rather than single wavelengths (Štrumbelj and Kononenko, 2014). By using a single-wavelength approach based on infinitesimal perturbations in Cui and Fearn (2018), the studied CNNs were observed to have increased robustness compared to PLS models. A similar single-wavelength perturbation approach was used in Schiemer et al. (2023) to perform automated variable selection for a Gaussian process regression model for monitoring an antibody-drug conjugation reaction.

Next to the identification of important features, removal-based methods such as SHAP may also be used to determine the sensitivity of specific features (Covert et al., 2020b) which could potentially be applied to detect failures in chemometric models. In the future, it would be interesting to employ such methods in real-time during the optimization or maintenance of chemometric models. When new data are available from a manufacturing process, individual feature contributions may be beneficial to identify potential process or model failures and hence trigger model updating mechanisms (Nikzad-Langerodi et al., 2018; Krause et al., 2021; FDA, 2023). In summary, we consider the usage of multiple orthogonal variable importance methods crucial to build explainable and robust chemometric models, and to ensure that the most relevant structural and chemical information present in the spectroscopic data are used for prediction.

### 4.4 Robustness

Spectral data are often corrupted by noise or baseline and peak shifts (Bjerrum et al., 2017; Blazhko et al., 2021). Routinely applied operations to counteract these effects are among others spectral smoothing, derivation, or corrections (Anderson et al., 2020; Tulsyan et al., 2021). Spectral data may contain different levels of noise or baseline effects due to new data being recorded with a different spectrometer (Mishra and Passos, 2021a), biological variability (Tulsyan et al., 2021) or as part of a technology transfer to another site (Christler et al., 2021).

To evaluate the robustness of CNNs compared to PLS models, an *in silico* noise perturbation study was performed. In summary, the CNNs were found to be more robust against increasing noise and wavelength shifts than PLS models. CNNs have been shown to be less susceptible to noise in images and electrocardiograms depending on the architecture used (Rodríguez-Rodríguez et al., 2021; Venton et al., 2021). In general, the increased robustness may be attributed to various features inherent to CNNs, such as convolution operations and in-built regularization. Each input is transformed by a convolution operation followed by a non-linear activation and a max-pooling operation. By stacking multiple convolutional blocks in sequence, a funnel-like structure is created which is considered to increase robustness. Secondly, regularization methods like dropout and weight regularization can lead to increased robustness (Krizhevsky et al., 2017). In chemometrics, Cui and Fearn (2018) have shown that using dropout reduced noise in the trained weights of their CNNs, while Mishra and Passos (2021a) could show that tuning the regularization factor led to improved performance on newly acquired data.

In summary, the CNNs used in this study appear to be more robust against different sorts of noise compared to PLS models, whereas it is not clear which of the mentioned aspects are the main contributors to the robustness of the model. It is thus suggested to evaluate the robustness of chemometric models in an independent study specifically designed for that purpose.

## 5 Conclusion

This study demonstrates the augmentation, optimization, and interpretation of CNNs for process monitoring using spectroscopic data. The herein proposed LSA data augmentation method was shown to generate realistic *in silico* spectra for three data sets based on UV/Vis spectroscopy of model proteins and mAb size variants, and one data set based on IR spectroscopy. By augmenting CNN models with the *in silico* data, the prediction accuracy for the detection of mAb size variants could be improved by up to 50% compared to conventional PLS models.

Through automated optimization, the neural network architecture and the configuration of other model elements could be simultaneously tuned to maximize model performance. The combined optimization led to neural network structures with multiple convolutional blocks similar to previously published models, while most of the accuracy boost could be attributed to the data augmentation approach for UV/Vis spectroscopy data. Transferring the optimized architecture without prior HPO to a data set with multiple mAb size variants resulted in comparable or superior accuracy compared to PLS models, proving good generalizability of the optimized CNN model. Although the CNN model in combination with the LSA data augmentation method provided promising results for four data sets, further studies are required to validate the applicability of the LSA method to cases where more background effects are present such as IR or Raman spectroscopy data. Finally, the optimized CNN and PLS models were directly compared with regard to the importance of different wavelength regions and robustness against spectral noise. While model-agnostic methods such as GradCAM and VIPs were able to provide specific importance estimates for the CNN and PLS models, respectively, SHAP was able to resolve differences between the two model types directly and suggested an improved capability to discriminate between mAb size-variants. Additionally, the studied CNNs appear to be more robust against synthetic white noise and peak shifts in the spectral data. However, this property should be evaluated in more detail with real data involving biological and spectral variability.

In summary, this study expands on previous works on CNNs for chemometrics and proves the applicability of the suggested methods for the quantification of model proteins and mAb size variants. The deployment of the demonstrated workflow is considered to improve the accuracy, generalizability, and scalability of chemometric models used for process monitoring and control.

## Data Availability

The raw data supporting the conclusion of this article will be made available by the authors, without undue reservation.

## References

[B1] Acquarelli J. van Laarhoven T. Gerretzen J. Tran T. N. Buydens L. M. Marchiori E. (2017). Convolutional neural networks for vibrational spectroscopic data analysis. Anal. Chim. Acta 954, 22–31. 10.1016/j.aca.2016.12.010 28081811

[B2] Afseth N. K. Kohler A. (2012). Extended multiplicative signal correction in vibrational spectroscopy, a tutorial. Chemom. Intelligent Laboratory Syst. 117, 92–99. 10.1016/j.chemolab.2012.03.004

[B3] Akiba T. Sano S. Yanase T. Ohta T. Koyama M. (2019). Optuna: a next-generation hyperparameter optimization framework. Proceedings of the ACM SIGKDD International Conference on Knowledge Discovery and Data Mining, Anchorage, AK, USA, August 4-8, 2019, 2623–2631. 10.1145/3292500.3330701

[B4] Anderson N. Walsh K. Subedi P. Hayes C. (2020). Achieving robustness across season, location and cultivar for a NIRS model for intact mango fruit dry matter content. Postharvest Biol. Technol. 168, 111202. 10.1016/j.postharvbio.2020.111202

[B5] Bakeev K. A. (2005). Process analytical technology. 1. Oxford, GB: Blackwell Publishing.

[B6] Banner M. Alosert H. Spencer C. Cheeks M. Farid S. S. Thomas M. (2021). A decade in review: use of data analytics within the biopharmaceutical sector. Curr. Opin. Chem. Eng. 34, 100758. 10.1016/j.coche.2021.100758 PMC8665905 34926134

[B7] Belle V. Papantonis I. (2021). Principles and practice of explainable machine learning. Front. Big Data 4, 1–25. 10.3389/fdata.2021.688969 PMC8281957 34278297

[B8] Bergstra J. Yamins D. Cox D. (2013). “Making a science of model search: hyperparameter optimization in hundreds of dimensions for vision architectures (JMLR.org),” in Proceedings of the 30th International Conference on Machine Learning, vol. 28, I–115–I–123, Atlanta, GA, USA, 16-21 June 2013.

[B9] Bjerrum E. J. Glahder M. Skov T. (2017). Data augmentation of spectral data for convolutional neural network (cnn) based deep chemometrics, 1–10. Available at: https://arxiv.org/abs/1710.01927.

[B10] Blazhko U. Shapaval V. Kovalev V. Kohler A. (2021). Comparison of augmentation and pre-processing for deep learning and chemometric classification of infrared spectra. Chemom. Intelligent Laboratory Syst. 215, 104367. 10.1016/j.chemolab.2021.104367

[B11] Brestrich N. Hahn T. Hubbuch J. (2016). Application of spectral deconvolution and inverse mechanistic modelling as a tool for root cause investigation in protein chromatography. J. Chromatogr. A 1437, 158–167. 10.1016/j.chroma.2016.02.011 26879457

[B12] Brestrich N. Rüdt M. Büchler D. Hubbuch J. (2018). Selective protein quantification for preparative chromatography using variable pathlength UV/Vis spectroscopy and partial least squares regression. Chem. Eng. Sci. 176, 157–164. 10.1016/j.ces.2017.10.030

[B13] Brunel B. Alsamad F. Piot O. (2021). Toward automated machine learning in vibrational spectroscopy - use and settings of genetic algorithms for pre-processing and regression optimization. Chemom. Intelligent Laboratory Syst. 219, 1–10. 10.1016/j.chemolab.2021.104444

[B14] Burkart N. Huber M. F. (2021). A survey on the explainability of supervised machine learning. J. Artif. Intell. Res. 70, 245–317. 10.1613/jair.1.12228

[B15] Capito F. Skudas R. Kolmar H. Stanislawski B. (2013). Host cell protein quantification by fourier transform mid infrared spectroscopy (ft-mir). Biotechnol. Bioeng. 110, 252–259. 10.1002/bit.24611 22811255

[B16] Christler A. Scharl T. Sauer D. G. Köppl J. Tscheließnig A. Toy C. (2021). Technology transfer of a monitoring system to predict product concentration and purity of biopharmaceuticals in real-time during chromatographic separation. Biotechnol. Bioeng. 118, 3941–3952. Publisher: John Wiley and Sons Inc. 10.1002/bit.27870 34170524 PMC8518415

[B17] Covert I. Lundberg S. Lee S.-I. (2020a). Explaining by removing: a unified framework for model explanation. J. Mach. Learn. Res. 22, 1–90.

[B18] Covert I. Lundberg S. Lee S.-I. (2020b). Understanding global feature contributions with additive importance measures. Advances in Neural Information Processing Systems 2020-Decem. Available at: https://arxiv.org/abs/2004.00668.

[B19] Cui C. Fearn T. (2017). Comparison of partial least squares regression, least squares support vector machines, and Gaussian process regression for a near infrared calibration. J. Near Infrared Spectrosc. 25, 5–14. 10.1177/0967033516678515

[B20] Cui C. Fearn T. (2018). Modern practical convolutional neural networks for multivariate regression: applications to nir calibration. Chemom. Intelligent Laboratory Syst. 182, 9–20. 10.1016/j.chemolab.2018.07.008

[B21] FDA (2004). Guidance for industry PAT - a framework for innovative pharmaceutical development, manufacuring, and quality assurance. Available at: http://www.fda.gov/downloads/Drugs/GuidanceComplianceRegulatoryInformation/Guidances/ucm070305.pdf.

[B22] FDA (2023). Artificial intelligence in drug manufacturing. Available at: https://www.fda.gov/media/165743/download.

[B23] Feidl G. Luna V. Souquet B. Vogg S. Souquet J. Broly H. (2019). Combining mechanistic modeling and Raman spectroscopy for monitoring antibody chromatographic purification. Processes 7, 683. 10.3390/pr7100683

[B24] Feng S. Y. Gangal V. Wei J. Chandar S. Vosoughi S. Mitamura T. (2021). A survey of data augmentation approaches for NLP. Find. Assoc. Comput. Linguistics ACL-IJCNLP 2021, 968–988. 10.18653/v1/2021.findings-acl.84

[B25] Feurer M. Hutter F. (2019). Hyperparameter optimization. Cham: Springer International Publishing, 3–33. 10.1007/978-3-030-05318-5_1

[B26] Glassey J. Gernaey K. V. Clemens C. Schulz T. W. Oliveira R. Striedner G. (2011). Process analytical technology (PAT) for biopharmaceuticals. Biotechnol. J. 6, 369–377. 10.1002/biot.201000356 21416609

[B27] Goldrick S. Umprecht A. Tang A. Zakrzewski R. Cheeks M. Turner R. (2020). High-throughput Raman spectroscopy combined with innovate data analysis workflow to enhance biopharmaceutical process development. Processes 8, 1179. 10.3390/pr8091179

[B28] Goodfellow I. J. Bengio Y. Courville A. (2016). Deep learning. Cambridge, MA, USA: MIT Press. Available at: http://www.deeplearningbook.org.

[B29] Gu J. Wang Z. Kuen J. Ma L. Shahroudy A. Shuai B. (2018). Recent advances in convolutional neural networks. Pattern Recognit. 77, 354–377. 10.1016/j.patcog.2017.10.013

[B30] Guindo M. L. Kabir M. H. Chen R. Huang J. Liu F. Li X. (2023). Chemometric approach based on explainable AI for rapid assessment of macronutrients in different organic fertilizers using fusion spectra. Molecules 28, 799. 10.3390/molecules28020799 36677857 PMC9862029

[B31] Guo F. Xie R. Huang B. (2020). A deep learning just-in-time modeling approach for soft sensor based on variational autoencoder. Chemom. Intelligent Laboratory Syst. 197, 103922. 10.1016/j.chemolab.2019.103922

[B32] Haghi R. K. Pérez-Fernández E. Robertson A. H. (2021). Prediction of various soil properties for a national spatial dataset of Scottish soils based on four different chemometric approaches: a comparison of near infrared and mid-infrared spectroscopy. Geoderma 396, 115071. 10.1016/j.geoderma.2021.115071

[B33] Hansen S. K. Skibsted E. Staby A. Hubbuch J. (2011). A label-free methodology for selective protein quantification by means of absorption measurements. Biotechnol. Bioeng. 108, 2661–2669. 10.1002/bit.23229 21755495

[B34] Jiang M. Severson K. A. Love J. C. Madden H. Swann P. Zang L. (2017). Opportunities and challenges of real-time release testing in biopharmaceutical manufacturing. Biotechnol. Bioeng. 114, 2445–2456. 10.1002/bit.26383 28710854

[B35] Kingma D. P. Ba J. L. (2015). “Adam: a method for stochastic optimization,” in 3rd International Conference on Learning Representations, ICLR 2015 - Conference Track Proceedings , 1–15, San Diego, CA, USA, May 7-9, 2015.

[B36] Krause J. Günder M. Schulz D. Gruna R. (2021). New active learning algorithms for near-infrared spectroscopy in agricultural applications. A. T. - Autom. 69, 297–306. 10.1515/auto-2020-0143

[B37] Krizhevsky A. Sutskever I. Hinton G. E. (2017). ImageNet classification with deep convolutional neural networks. Commun. ACM 60, 84–90. 10.1145/3065386

[B38] Li M.-Y. Ebel B. Paris C. Chauchard F. Guedon E. Marc A. (2018). Real-time monitoring of antibody glycosylation site occupancy by *in situ* Raman spectroscopy during bioreactor cho cell cultures. Biotechnol. Prog. 34, 486–493. 10.1002/btpr.2604 29314747

[B39] Liu J. Osadchy M. Ashton L. Foster M. Solomon C. J. Gibson S. J. (2017). Deep convolutional neural networks for Raman spectrum recognition: a unified solution. Analyst 142, 4067–4074. 10.1039/c7an01371j 28993828

[B40] Long J. R. Gregoriou V. G. Gemperline P. J. (1990). Spectroscopic calibration and quantitation using artificial neural networks. Anal. Chem. 62, 1791–1797. 10.1021/ac00216a013

[B41] Lundberg S. M. Lee S.-I. (2017). “A unified approach to interpreting model predictions,” in Advances in neural information processing systems 30. Editors Guyon I. Luxburg U. V. Bengio S. Wallach H. Fergus R. Vishwanathan S. (Red Hook, New York, United States: Curran Associates, Inc.), 4765–4774. Available at: https://proceedings.neurips.cc/paper_files/paper/2017/file/8a20a8621978632d76c43dfd28b67767-Paper.pdf

[B42] Mahynski N. A. Ragland J. M. Schuur S. S. Shen V. K. (2022). Building interpretable machine learning models to identify chemometric trends in seabirds of the north pacific ocean. Environ. Sci. Technol. 56, 14361–14374. 10.1021/acs.est.2c01894 36197753

[B43] Malek S. Melgani F. Bazi Y. (2018). One-dimensional convolutional neural networks for spectroscopic signal regression. J. Chemom. 32, 1–17. 10.1002/cem.2977

[B44] Markl D. Warman M. Dumarey M. Bergman E.-L. Folestad S. Shi Z. (2020). Review of real-time release testing of pharmaceutical tablets: state-of-the art, challenges and future perspective. Int. J. Pharm. 582, 119353. 10.1016/j.ijpharm.2020.119353 32325242

[B45] Martens H. Stark E. (1991). Extended multiplicative signal correction and spectral interference subtraction: new preprocessing methods for near infrared spectroscopy. J. Pharm. Biomed. Analysis 9, 625–635. 10.1016/0731-7085(91)80188-F 1790182

[B46] McHardy G. Antoniou G. Conn A. Baker J. Palmer S. (2023). Augmentation of FTIR spectral datasets using Wasserstein generative adversarial networks for cancer liquid biopsies. Analyst 148, 3860–3869. Publisher: Royal Society of Chemistry. 10.1039/D3AN00669G 37435822

[B47] Mehmood T. Liland K. H. Snipen L. Sæbø S. (2012). A review of variable selection methods in partial least squares regression. Chemom. Intelligent Laboratory Syst. 118, 62–69. 10.1016/j.chemolab.2012.07.010

[B48] Mishra P. Herrmann I. (2021). Gan meets chemometrics: segmenting spectral images with pixel2pixel image translation with conditional generative adversarial networks. Chemom. Intelligent Laboratory Syst. 215, 104362. 10.1016/j.chemolab.2021.104362

[B49] Mishra P. Passos D. (2021a). Deep chemometrics: validation and transfer of a global deep near-infrared fruit model to use it on a new portable instrument. J. Chemom. 35, e3367. 10.1002/cem.3367

[B50] Mishra P. Passos D. (2021b). Realizing transfer learning for updating deep learning models of spectral data to be used in new scenarios. Chemom. Intelligent Laboratory Syst. 212, 104283. 10.1016/j.chemolab.2021.104283

[B51] Mishra P. Passos D. (2021c). Realizing transfer learning for updating deep learning models of spectral data to be used in new scenarios. Chemom. Intelligent Laboratory Syst. 212, 104283. 10.1016/j.chemolab.2021.104283

[B52] Mishra P. Passos D. (2021d). A synergistic use of chemometrics and deep learning improved the predictive performance of near-infrared spectroscopy models for dry matter prediction in mango fruit. Chemom. Intelligent Laboratory Syst. 212, 104287. 10.1016/j.chemolab.2021.104287

[B53] Mishra P. Roger J. M. Marini F. Biancolillo A. Rutledge D. N. (2021). Parallel pre-processing through orthogonalization (porto) and its application to near-infrared spectroscopy. Chemom. Intelligent Laboratory Syst. 212, 104190. 10.1016/j.chemolab.2020.104190

[B54] Nikzad-Langerodi R. Lughofer E. Cernuda C. Reischer T. Kantner W. Pawliczek M. (2018). Calibration model maintenance in melamine resin production: integrating drift detection, smart sample selection and model adaptation. Anal. Chim. Acta 1013, 1–12. 10.1016/j.aca.2018.02.003 29501087

[B55] Passos D. Mishra P. (2021). An automated deep learning pipeline based on advanced optimisations for leveraging spectral classification modelling. Chemom. Intelligent Laboratory Syst. 215, 104354. 10.1016/j.chemolab.2021.104354

[B56] Passos D. Mishra P. (2022). A tutorial on automatic hyperparameter tuning of deep spectral modelling for regression and classification tasks. Chemom. Intelligent Laboratory Syst. 223, 104520. 10.1016/j.chemolab.2022.104520

[B57] Read E. Park J. Shah R. Riley B. Brorson K. Rathore A. (2010a). Process analytical technology (pat) for biopharmaceutical products: Part i. concepts and applications. Biotechnol. Bioeng. 105, 276–284. 10.1002/bit.22528 19731252

[B58] Read E. K. Shah R. B. Riley B. S. Park J. T. Brorson K. A. Rathore A. S. (2010b). Process Analytical Technology (PAT) for biopharmaceutical products: Part II. Concepts and applications. Biotechnol. Bioeng. 105, 285–295. 10.1002/bit.22529 19731253

[B59] Rodríguez-Rodríguez J. A. Molina-Cabello M. A. Benítez-Rochel R. López-Rubio E. (2021). “The impact of linear motion blur on the object recognition efficiency of deep convolutional neural networks,” in Pattern recognition. ICPR international workshops and challenges. Editors Del Bimbo A. Cucchiara R. Sclaroff S. Farinella G. M. Mei T. Bertini M. (Cham: Springer International Publishing), Lecture Notes in Computer Science), 611–622. 10.1007/978-3-030-68780-9_47

[B60] Rolinger L. Hubbuch J. Rüdt M. (2023). Monitoring of ultra- and diafiltration processes by Kalman-filtered Raman measurements. Anal. Bioanal. Chem. 415, 841–854. 10.1007/s00216-022-04477-7 36651972 PMC9883314

[B61] Rolinger L. Rüdt M. Hubbuch J. (2020). A critical review of recent trends, and a future perspective of optical spectroscopy as PAT in biopharmaceutical downstream processing. Anal. Bioanal. Chem. 412, 2047–2064. 10.1007/s00216-020-02407-z 32146498 PMC7072065

[B62] Rolinger L. Rüdt M. Hubbuch J. (2021). Comparison of uv- and Raman-based monitoring of the protein a load phase and evaluation of data fusion by pls models and cnns. Biotechnol. Bioeng. 118, 4255–4268. 10.1002/bit.27894 34297358

[B63] Romann P. Kolar J. Tobler D. Herwig C. Bielser J. M. Villiger T. K. (2022). Advancing Raman model calibration for perfusion bioprocesses using spiked harvest libraries. Biotechnol. J. 17, e2200184. 10.1002/biot.202200184 35900328

[B64] Rosebrock A. (2018). Deep learning for computer vision with Python (pyimagesearch).

[B65] Rüdt M. Andris S. Schiemer R. Hubbuch J. (2019). Factorization of preparative protein chromatograms with hard-constraint multivariate curve resolution and second-derivative pretreatment. J. Chromatogr. A 1585, 152–160. 10.1016/j.chroma.2018.11.065 30528712

[B66] Rüdt M. Brestrich N. Rolinger L. Hubbuch J. (2017a). Real-time monitoring and control of the load phase of a protein a capture step. Biotechnol. Bioeng. 114, 368–373. 10.1002/bit.26078 27543789 PMC5215519

[B67] Rüdt M. Briskot T. Hubbuch J. (2017b). Advances in downstream processing of biologics – spectroscopy: an emerging process analytical technology. J. Chromatogr. A 1490, 2–9. 10.1016/j.chroma.2016.11.010 27887700

[B68] Sanden A. Suhm S. Rüdt M. Hubbuch J. (2019). Fourier-transform infrared spectroscopy as a process analytical technology for near real time in-line estimation of the degree of pegylation in chromatography. J. Chromatogr. A 1608, 460410. 10.1016/j.chroma.2019.460410 31395360

[B69] Santos V. O. Oliveira F. C. Lima D. G. Petry A. C. Garcia E. Suarez P. A. (2005). A comparative study of diesel analysis by ftir, ftnir and ft-Raman spectroscopy using pls and artificial neural network analysis. Anal. Chim. Acta 547, 188–196. 10.1016/j.aca.2005.05.042

[B70] Sauer D. G. Melcher M. Mosor M. Walch N. Berkemeyer M. Scharl-Hirsch T. (2019). Real-time monitoring and model-based prediction of purity and quantity during a chromatographic capture of fibroblast growth factor 2. Biotechnol. Bioeng. 116, 1999–2009. 10.1002/bit.26984 30934111 PMC6618329

[B71] Schiemer R. Weggen J. T. Schmitt K. M. Hubbuch J. (2023). An adaptive soft-sensor for advanced real-time monitoring of an antibody-drug conjugation reaction. Biotechnol. Bioeng. 120, 1914–1928. 10.1002/bit.28428 37190793

[B72] Selvaraju R. R. Cogswell M. Das A. Vedantam R. Parikh D. Batra D. (2020). Grad-cam: visual explanations from deep networks via gradient-based localization. Int. J. Comput. Vis. 128, 336–359. 10.1007/s11263-019-01228-7

[B73] Shorten C. Khoshgoftaar T. M. (2019). A survey on image data augmentation for deep learning. J. Big Data 6, 60. 10.1186/s40537-019-0197-0 PMC8287113 34306963

[B74] Štrumbelj E. Kononenko I. (2014). Explaining prediction models and individual predictions with feature contributions. Knowl. Inf. Syst. 41, 647–665. 10.1007/s10115-013-0679-x

[B75] Szegedy C. Liu W. Jia Y. Sermanet P. Reed S. Anguelov D. (2015). Going deeper with convolutions. In 2015 IEEE Conference on Computer Vision and Pattern Recognition (CVPR). 1–9. Boston, MA, USA, 7-12 June 2015. 10.1109/CVPR.2015.7298594

[B76] Trampuž M. Teslić D. Likozar B. (2020). Process analytical technology-based (pat) model simulations of a combined cooling, seeded and antisolvent crystallization of an active pharmaceutical ingredient (api). Powder Technol. 366, 873–890. 10.1016/j.powtec.2020.03.027

[B77] Tulsyan A. Garvin C. Undey C. (2019). Industrial batch process monitoring with limited data. J. Process Control 77, 114–133. 10.1016/j.jprocont.2019.03.002

[B78] Tulsyan A. Khodabandehlou H. Wang T. Schorner G. Coufal M. Undey C. (2021). Spectroscopic models for real-time monitoring of cell culture processes using spatiotemporal just-in-time Gaussian processes. AIChE J. 67. 10.1002/aic.17210

[B79] Ündey C. Ertun S. Mistretta T. Looze B. (2010). Applied advanced process analytics in biopharmaceutical manufacturing: challenges and prospects in real-time monitoring and control. J. Process Control 20, 1009–1018. 10.1016/j.jprocont.2010.05.008

[B80] Venton J. Harris P. M. Sundar A. Smith N. A. S. Aston P. J. (2021). Robustness of convolutional neural networks to physiological electrocardiogram noise. Philosophical Trans. R. Soc. A Math. Phys. Eng. Sci. 379, 20200262. 10.1098/rsta.2020.0262 PMC8543045 34689617

[B81] Wang B. Bowles-Welch A. C. Yeago C. Roy K. (2022). Process analytical technologies in cell therapy manufacturing: state-of-the-art and future directions. J. Adv. Manuf. Process. 4, 1–17. 10.1002/amp2.10106

[B82] Wang J. Chen J. Studts J. Wang G. (2023). In-line product quality monitoring during biopharmaceutical manufacturing using computational Raman spectroscopy. mAbs 15, 2220149. 10.1080/19420862.2023.2220149 37288839 PMC10251777

[B83] Wegner C. H. Hubbuch J. (2022). Calibration-free pat: locating selective crystallization or precipitation sweet spot in screenings with multi-way parafac models. Front. Bioeng. Biotechnol. 10, 1–18. 10.3389/fbioe.2022.1051129 PMC9797130 36588941

[B84] Wei B. Woon N. Dai L. Fish R. Tai M. Handagama W. (2022). Multi-attribute Raman spectroscopy (mars) for monitoring product quality attributes in formulated monoclonal antibody therapeutics. mAbs 14, 2007564. 10.1080/19420862.2021.2007564 34965193 PMC8726703

[B85] Wold S. Sjöström M. Eriksson L. (2001). Pls-regression: a basic tool of chemometrics. Chemom. Intelligent Laboratory Syst. 58, 109–130. 10.1016/S0169-7439(01)00155-1

[B86] Wu M. Wang S. Pan S. Terentis A. C. Strasswimmer J. Zhu X. (2021). Deep learning data augmentation for Raman spectroscopy cancer tissue classification. Sci. Rep. 11, 23842. Number: 1 Publisher: Nature Publishing Group. 10.1038/s41598-021-02687-0 34903743 PMC8668947

[B87] Yosinski J. Clune J. Nguyen A. Fuchs T. Lipson H. (2015). Understanding neural networks through deep visualization. Available at: https://arxiv.org/abs/1506.06579.

[B88] Yuanyuan C. Zhibin W. (2018). Quantitative analysis modeling of infrared spectroscopy based on ensemble convolutional neural networks. Chemom. Intelligent Laboratory Syst. 181, 1–10. 10.1016/j.chemolab.2018.08.001

[B89] Zeiler M. D. Fergus R. (2013). “Visualizing and understanding convolutional networks,” in Lecture notes in computer science (including subseries lecture notes in artificial intelligence and lecture notes in bioinformatics) 8689 LNCS, 818–833. Visualization of imageNet convolutional layers by deconvolutional networks applied to the trained model to project the filter down to pixel space and show e.g the strongest activation patterns. 10.1007/978-3-319-10590-1_53

[B90] Zhang C. Springall J. S. Wang X. Barman I. (2019a). Rapid, quantitative determination of aggregation and particle formation for antibody drug conjugate therapeutics with label-free Raman spectroscopy. Anal. Chim. Acta 1081, 138–145. 10.1016/j.aca.2019.07.007 31446951 PMC6750807

[B91] Zhang X. Lin T. Xu J. Luo X. Ying Y. (2019b). Deepspectra: an end-to-end deep learning approach for quantitative spectral analysis. Anal. Chim. Acta 1058, 48–57. 10.1016/j.aca.2019.01.002 30851853

