# Supplementary material for: Generative data augmentation and automated optimization of convolutional neural networks for process monitoring
Source: Front Bioeng Biotechnol. 2024 Jan 31;12:1228846. doi: 10.3389/fbioe.2024.1228846 (PMC10864647; doi:10.3389/fbioe.2024.1228846)
Supplement: Supplementary file 1 [file DataSheet1.PDF]

Supplementary Material

1 SUBSET SELECTION FOR DATA AUGMENTATION

This section provides additional information on the subset selection performed during data augmentation. For clarity, we provide a simple example which may directly be transferred to any case dealt with in this study. Let the data set contain 100 training samples at concentrations from 0 to $5\,\mathrm{g\,L^{-1}}$ for component 1 and from 0 to $10\,\mathrm{g\,L^{-1}}$ for component 2. The following is a stepwise explanation of the LSA method:

1. **Estimation of the concentration distributions** The KDE aims to approximate the concentration distribution in the experimental data set. This is done using Gaussian kernels which are summed to obtain a multimodal Gaussian distribution. Figuratively, the experimentally obtained concentration values are plotted as a histogram. The KDE tries to approximate the histogram by fitting multiple Gaussian distributions. We compared the KDE approach with a uniform and a unimodal Gaussian distribution by training convolutional neural networks on the augmented data sets generated with the three distribution types. As the experimental data set only consists of discrete points with the most samples having low to medium protein concentration, the uniform and unimodal Gaussian approximation of the experimental data are thought to oversample regions where only few experimental data points are available. Analogously, the KDE approach is considered to only sample in similar concentration regions as provided in the experimental data.
2. **Sampling of a new concentration vector** From the estimated concentration distributions, a random sample is drawn. In our example, let this point have the concentrations of 1 and $2\,\mathrm{g\,L^{-1}}$ for components 1 and 2, respectively.
3. **Computation of the vector distance** The vector distances between the new concentration vector for which a new spectrum is to be generated and all concentration vectors (i.e. all samples in the training set) are computed. For an exemplary concentration vector from the training data with the concentrations 3 and $5\,\mathrm{g\,L^{-1}}$ for components 1 and 2, respectively, the respective vector distance is given by Equation (1) in the manuscript and yields $\sqrt{(1-3)^2+(2-5)^2}$ if we are using the Euclidean distance. This is repeated for all 100 data points in the training data.
4. **Selection of a subset** The vector distances are sorted in ascending order and a subset is formed by the n_{LSA} data points with smallest distance, effectively grouping together data points with similar concentrations.
5. **Generation of the *in silico* spectra** From the selected subset, the pure component profiles are estimated by solving Equation (2) in the manuscript. Using Equation (3) in the manuscript, the pure component profiles are recombined into a new *in silico* spectrum for the sampled concentration vector. Steps 2 to 5 are repeated until the number of desired augmented data points is reached.

2 PRACTICAL REMARKS FOR HYPERPARAMETER OPTIMIZATION

Although HPO facilitates automated tuning of ML models, it is important to mention that several prerequisites should be fulfilled when starting the optimization: (1) The objective function should be representative of the modeling objective. The herein used sum of coefficients of determination ensure equal weighting of all components which could similarly be achieved by other forms of normalization. (2) The configuration of the optimization algorithm was found to be crucial in determining a globally optimal

solution to not end up in premature, local minima of the objective function. The random sampling employed in the first 100 trials ensured that the search space was explored without any bias from initially sampled trials. Alternatively, the TPE sampler can be adjusted to balance between exploration and exploitation of the search spaces to achieve this. (3) In accordance with (1), it should further be ensured that all monitored optimization metrics are representative of one another such that e.g. the cross-validation error portrays the training or validation error in order to be able to guide to optimization towards a model capable of generalization. Especially, when pruning is used, the pruning metric should be selected to resemble the actual optimization metric. And lastly (4), it is advisable to conduct a preceding parameter study for selecting critical hyperparameters to be optimized. Adding more hyperparameters to the optimization problem, considerably increases the search space and thus may require longer optimization periods.

3 SUPPLEMENTARY TABLES AND FIGURES

3.1 Tables

Table S1. Overview of experimental conditions for data set 1 and 2.

Data set	Experiment	Elution Conditions	Number of samples	Subset
1	Run 1	250-400 mM NaCl (3 CV)	60	Training
1	Run 2	150-600 mM NaCl (3 CV)	56	Training
1	Run 3	230-460 mM NaCl (3 CV)	57	Training
1	Run 4	170-540 mM NaCl (3 CV)	60	Training
1	Run 5	200-500 mM NaCl (3 CV)	57	Test
2	Run 1	4 CV	101	Training
2	Run 2	5 CV	119	Test
2	Run 3	6 CV	139	Training
2	Run 4	7 CV	192	Training
3	Run 1	10 CV	72	Training
3	Run 2	20 CV	112	Test
3	Run 3	30 CV	164	Training
4	Randomly assigned	-	64	Training
4	Randomly assigned	-	16	Test

Table S2. Optimized PLS hyperparameters for data sets 1-4. All data were preprocessed using a SGF and mean-centering before being passed to the PLS models. For data sets 1-3, the number of components, the SGF window size and the order of derivative were optimized using cross-validated grid-based search in the ranges 2-15, 3-31 and 0-2 for the number of components, the SGF window and the order of derivative, respectively. For data set 4, solely the number of components was optimized in the range of 2-15.

Hyperparameter	Data set 1			Data set 2		Data set 3				Data set 4		
	Rib A	Cyt C	Lys	Monomer	Aggregate	LMWS1	Monomer	HMWS1	HMWS2	Oil	Protein	Starch
number of components	5	7	7	4	9	11	2	4	7	14	5	6
SGF window	17	3	9	21	3	9	3	7	11	19	19	19
order of derivative	2	1	2	0	0	1	2	2	2	2	2	2

Table S3. Comparison of LSA and EMSA method for data set 2 and 3 as measured by the RMSE for all components.

		LSA				EMSA				PLS			
		Training		Test		Training		Test		Training		Test	
	Component	RMSE	R2	RMSE	R2	RMSE	R2	RMSE	R2	RMSE	R2	RMSE	R2
Data set 2	monomer	1.044	0.990	1.1811	0.991	1.727	0.973	2.487	0.958	1.099	0.989	1.223	0.99
	aggregate	0.175	0.978	0.1903	0.98	0.320	0.927	0.370	0.923	0.411	0.879	0.494	0.862
Data set 4	oil	0.079	0.805	0.070	0.808	0.017	0.991	0.108	0.546	0.036	0.960	0.098	0.630
	protein	0.143	0.915	0.138	0.927	0.044	0.992	0.171	0.890	0.135	0.924	0.121	0.945
	starch	0.355	0.821	0.453	0.532	0.072	0.992	0.291	0.806	0.301	0.871	0.290	0.807

3.2 Figures

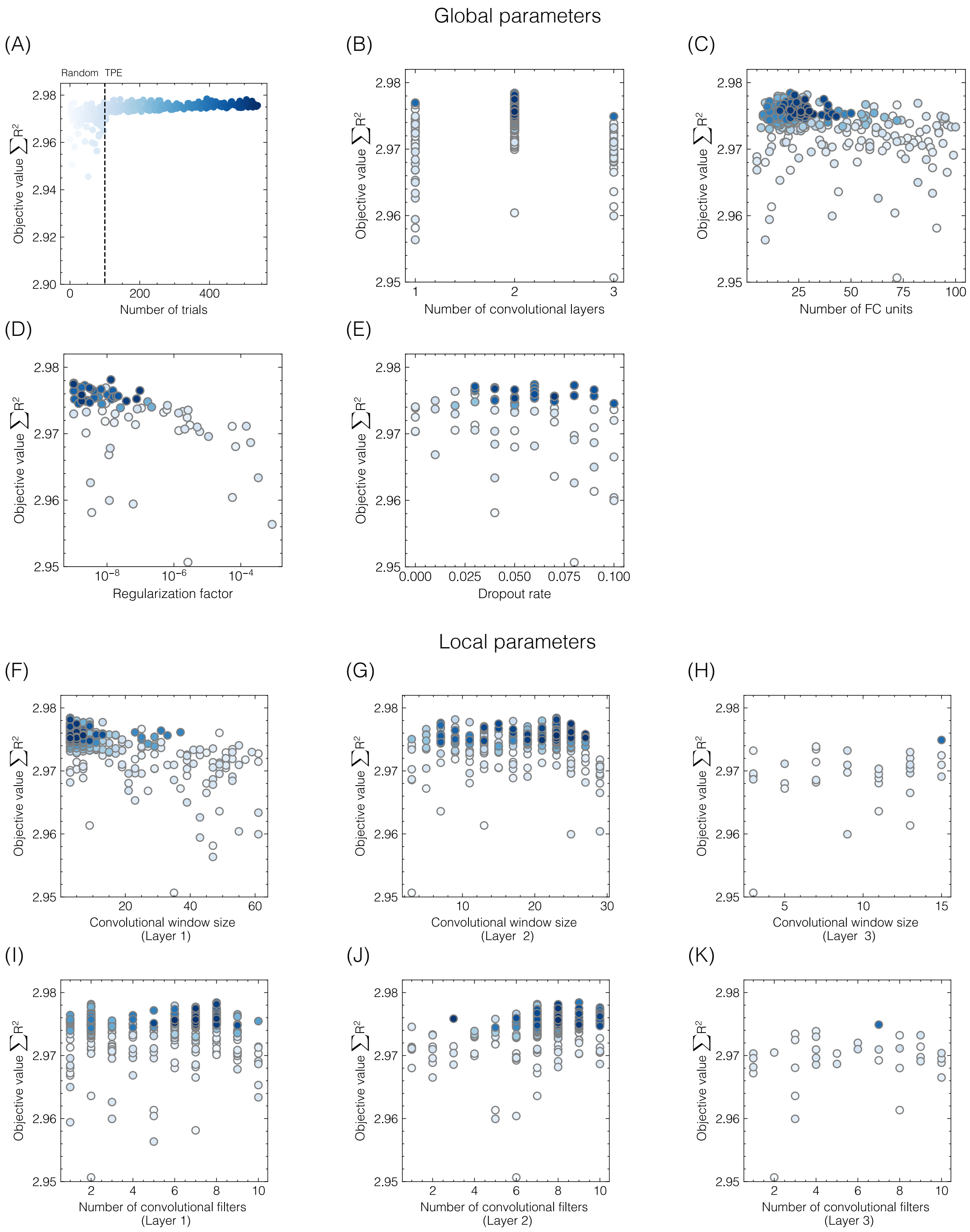

Figure S1. Hyperparameter evolution profiles during optimization for data set 1. The objective values $\sum R^2$ are shown for all trials (A) and each hyperparameter individually (B – K). The colors of the circles indicate the number of HPO trials with darker shades of blue corresponding to later sampling points. Random sampling was performed for the first 100 trials after which median pruning and the TPE-based optimization were enabled.

(A)

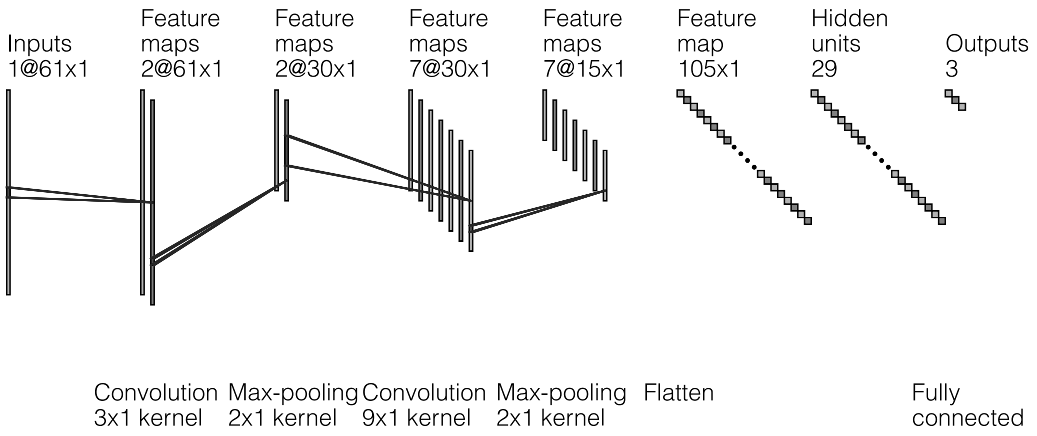

(B)

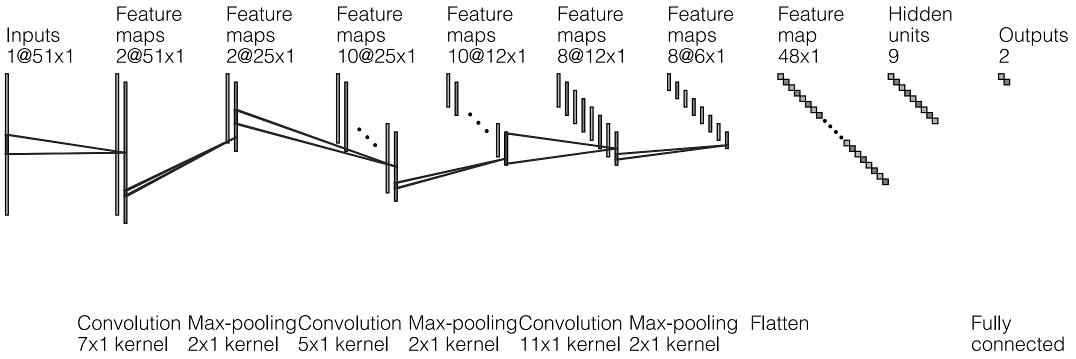

Figure S2. Visualization of the architecture of the optimized CNN models for data set 1 (A) and data set 2 (B). Spectral data being passed through the convolutional and pooling layers are shown as rectangles. The convolutional window is illustrated by the connecting lines. The exact dimensions of the individual layers and their respective inputs and outputs are given as $n_{\text{filters}}@d_{\text{spectrum}}$ as part of the labels above. The dimension of the convolution and pooling operations are given as part of the labels below. For each convolution operation, a number of filters n_{filters} are used.

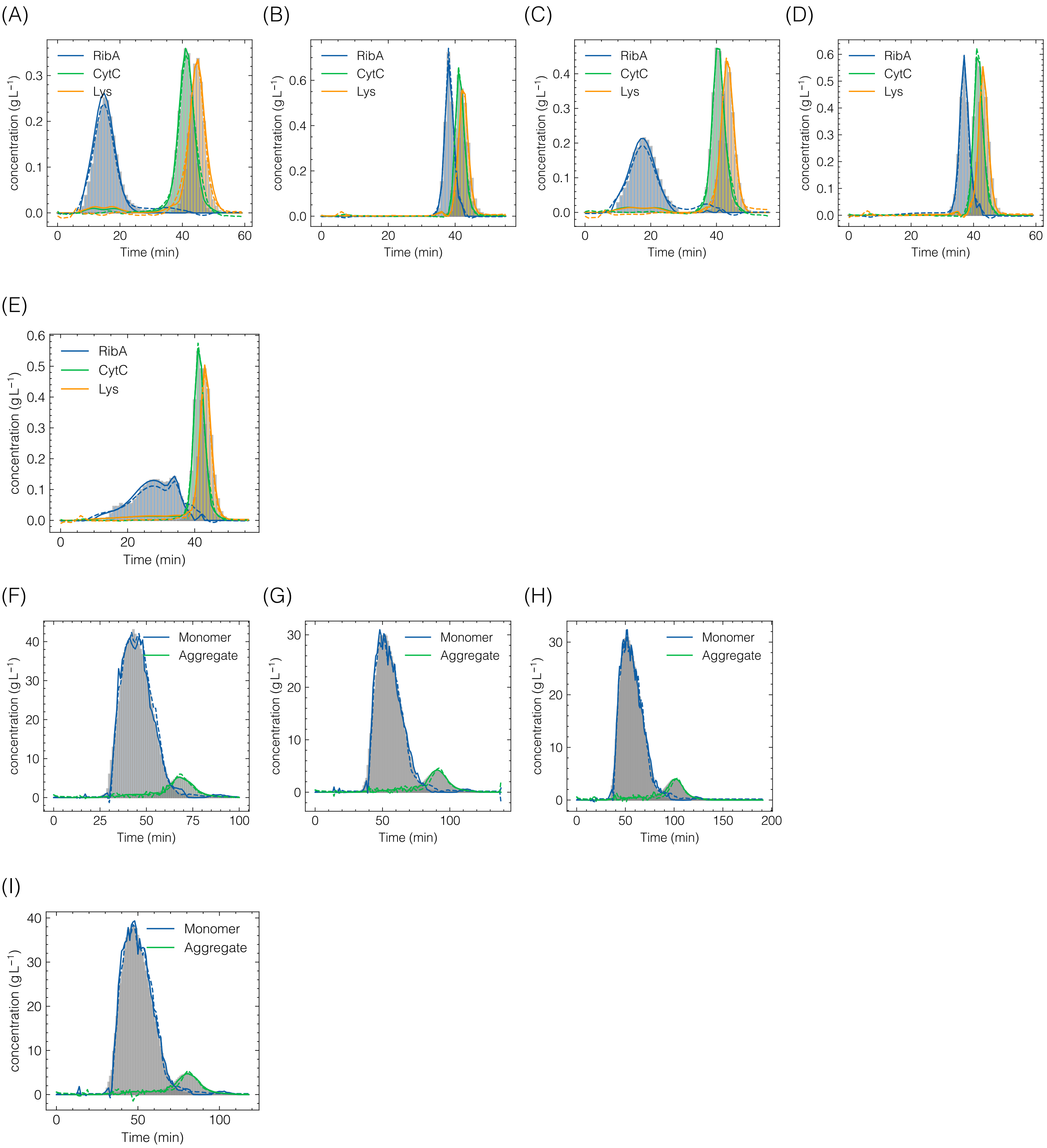

Figure S3. Predictions of the optimized CNN models. For data set 1, the training runs are shown in (A-D) and the test run is shown in (E). For data set 2, the training runs are shown in (F-H) and the test run in (H). The reference data is overlayed with the predicted values for all species as indicated by the bars and the solid lines, respectively. The PLS predictions are shown as dashed lines.

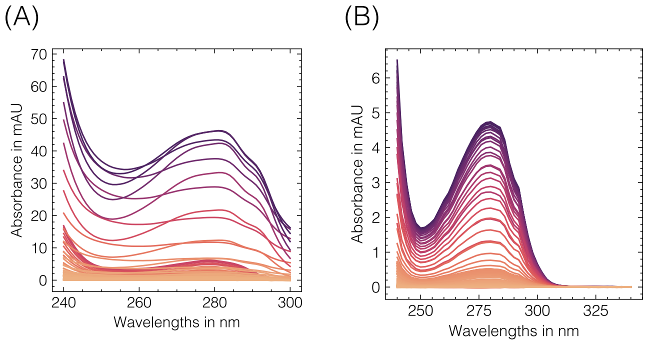

Figure S4. Exemplary raw UV/Vis spectra for data set 1 (A) and data set 2 (B). Run 1 and Run 3 are exemplarily shown for data set 1 and 2, respectively. The spectra are colored according to the total protein concentration in each sample with darker colors corresponding to higher concentration.

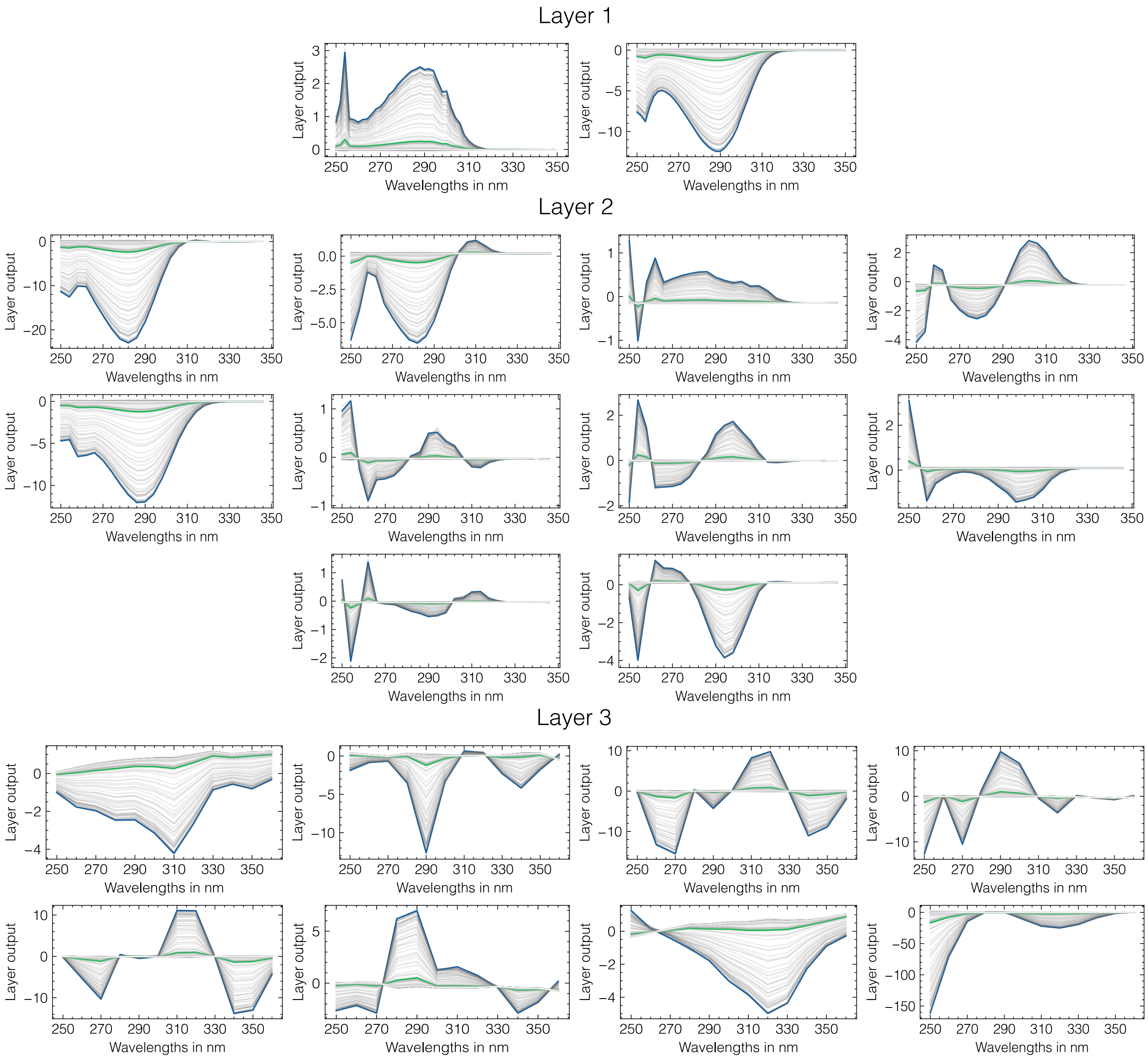

Figure S5. Layer visualization for all convolutional layer for the optimized CNN model for data set 2. For each convolutional filter, the outputs for all available spectra in the test subset are shown as gray lines. The spectra with the highest reference concentration for monomer and aggregate species are marked in blue and green, respectively. The dimensions of the outputs are reduced by a factor of 2 due to interposed pooling layers causing the resolution of the displayed lines to decrease.

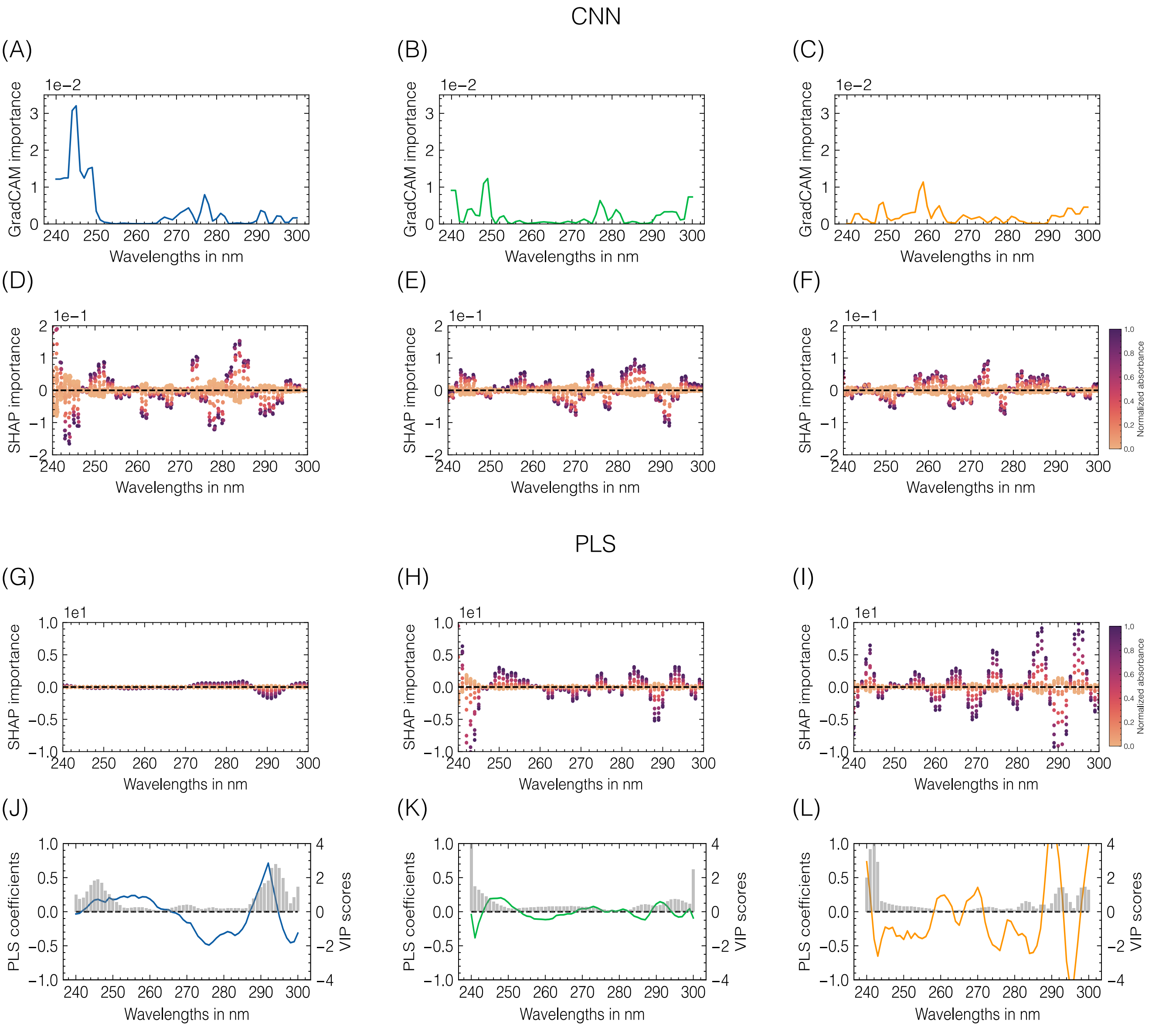

Figure S6. Model-specific and model-agnostic importance measures for data set 1. CNN-specific GradCAM importance values (A-C), SHAP values for CNN (D-F) and PLS (G-I) and PLS-specific regression coefficients and VIP scores (J-L). The regression coefficients and VIP scores are shown as solid lines and bars, respectively. The left, middle and right columns correspond to importance metrics for Rib A, Cyt C and Lys, respectively.

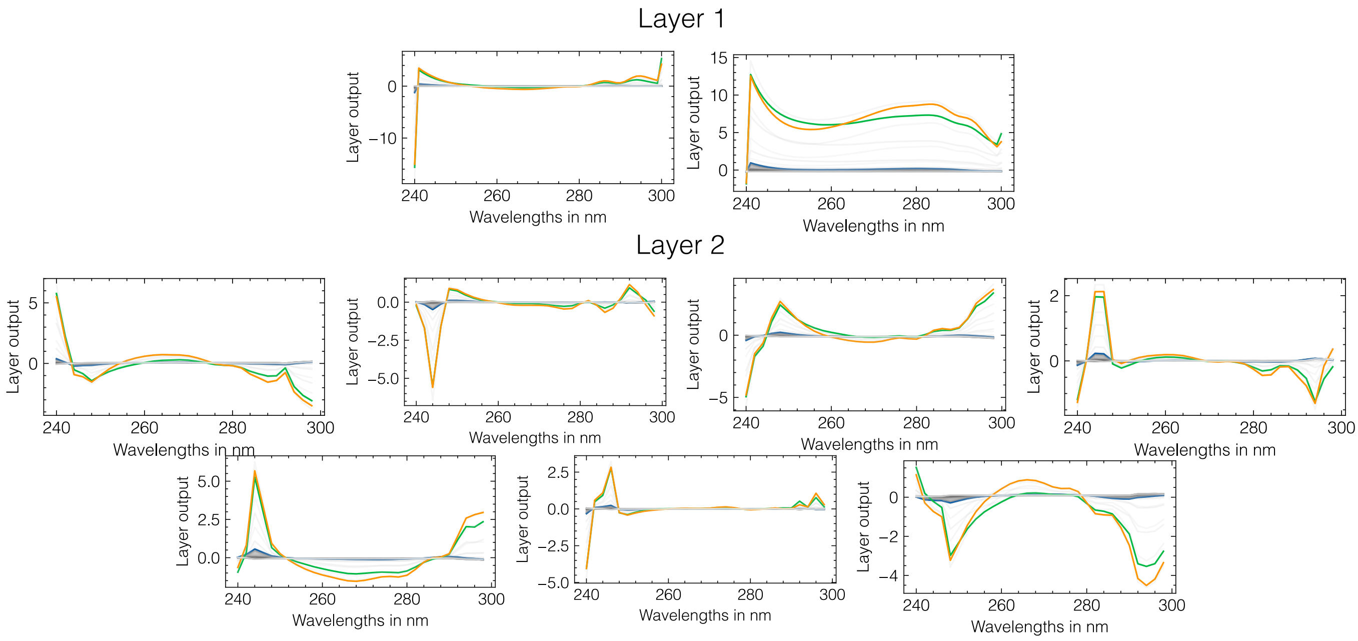

Figure S7. Layer visualization for all convolutional layer for the optimized CNN model for data set 1. For each convolutional filter, the outputs for all available spectra in the test subset are shown as gray lines. The spectra with the highest reference concentration for Rib A, Cyt C and Lys are marked in blue, green and orange, respectively. The dimensions of the outputs are reduced by a factor of 2 due to interposed pooling layers causing the resolution of the displayed lines to decrease.
